# Clinicopathological Implications of the *BRAF*^*V*600*E*^ Mutation in Papillary Thyroid Carcinoma of Ukrainian Patients Exposed to the Chernobyl Radiation in Childhood: A Study for 30 Years After the Accident

**DOI:** 10.3389/fmed.2022.882727

**Published:** 2022-04-26

**Authors:** Liudmyla Zurnadzhy, Tetiana Bogdanova, Tatiana I. Rogounovitch, Masahiro Ito, Mykola Tronko, Shunichi Yamashita, Norisato Mitsutake, Michael Bolgov, Serhii Chernyshov, Sergii Masiuk, Vladimir A. Saenko

**Affiliations:** ^1^Laboratory of Morphology of Endocrine System, State Institution “VP Komisarenko Institute of Endocrinology and Metabolism of the National Academy of Medical Sciences of Ukraine”, Kyiv, Ukraine; ^2^Department of Radiation Molecular Epidemiology, Atomic Bomb Disease Institute, Nagasaki University, Nagasaki, Japan; ^3^Department of Radiation Medical Sciences, Atomic Bomb Disease Institute, Nagasaki University, Nagasaki, Japan; ^4^Department of Diagnostic Pathology, National Hospital Organization Nagasaki Medical Center, Omura, Japan; ^5^Department of Fundamental and Applied Problems of Endocrinology, State Institution “VP Komisarenko Institute of Endocrinology and Metabolism of the National Academy of Medical Sciences of Ukraine”, Kyiv, Ukraine; ^6^Fukushima Medical University, Fukushima, Japan; ^7^National Institute of Radiological Sciences, National Institutes for Quantum Science and Technology, Chiba, Japan; ^8^Department of Surgery of Endocrine Glands, State Institution “VP Komisarenko Institute of Endocrinology and Metabolism of the National Academy of Medical Sciences of Ukraine”, Kyiv, Ukraine; ^9^Radiation Protection Laboratory, State Institution “National Research Center of Radiation Medicine of the National Academy of Medical Science of Ukraine”, Kyiv, Ukraine

**Keywords:** papillary thyroid carcinoma, Chernobyl accident, radiation, pathology, immunohistochemistry, BRAF^V600E^, Ki67

## Abstract

With time after the Chernobyl accident, the number of papillary thyroid carcinomas (PTCs) driven by the BRAF^V600E^ oncoprotein is growing in patients exposed to radiation at a young age. Clinicopathological associations of BRAF^V600E^ in PTCs from patients with internal radiation history have not been sufficiently studied so far. This work analyzes the structural characteristics, proliferative activity, invasive features, clinical information, and dosimetric data in the BRAF^V600E^-positive and BRAF^V600E^-negative PTCs from the Ukrainian patients exposed to Chernobyl radiation and treated over 30 years after the accident. The study included 428 PTCs from patients aged 4–49 years at surgery who lived in the six northern regions of Ukraine most contaminated by ^131^I, were ≤18 years of age at the time of exposure, and were operated on from 1990 to 2017. Immunohistochemical staining for BRAF^V600E^ was performed with the VE1 antibody. The probability of causation (POC) of a tumor due to radiation was determined using an interactive online NIH/NCI software. BRAF^V600E^ was detected in 136/428 (31.8%) PTCs. In comparison with the BRAF^V600E^-negative PTCs, the BRAF^V600E^-positivity was associated with older patient age at the accident and at surgery, a longer period of latency, and lower POC. The BRAF^V600E^-positive PTCs were characterized by smaller tumor size, higher Ki67 labeling index, more frequent oncocytic changes, multifocality, and dominant papillary growth pattern. Tumor invasive features were less frequent in the BRAF^V600E^-positive PTCs and did not change with POC level. Despite a less aggressive tumor phenotype, BRAF^V600E^ was a risk factor for recurrence, namely radioiodine-refractory (RAI-R) recurrent metastases. Multivariate models of RAI-R included BRAF^V600E^ and/or histopathological parameters closely correlating with BRAF^V600E^ such as tumor size, multifocality, dominant papillary growth pattern, or oncocytic changes. Thus, the BRAF^V600E^-positive PTCs from patients from a high-risk group for radiogenic thyroid cancer diagnosed in the 30 years after the Chernobyl accident did not display higher invasiveness regardless of POC level, but in view of the prognostic impact of this genetic alteration, knowledge of the BRAF status may be beneficial for middle-aged patients with radiogenic PTC considered for RAI therapy, and suggests more careful follow-up of patients with the BRAF^V600E^-positive tumors.

## Introduction

More than 35 years have passed since the Chernobyl accident whose major health effect on the exposed population has been an increase in the incidence of thyroid cancer. Children and adolescents born in 1968–1986 (i.e., aged up to 18 at the time of exposure) have the highest risk of developing radiogenic thyroid cancer (1); from 2006 all of them reached adult age (≥19 years old) at the time of possible surgery. Numerous publications of the previous years on epidemiology, histopathology, and molecular genetics of Chernobyl thyroid cancer, principally, the papillary thyroid carcinoma (PTC), usually involved the youngest exposed subjects operated on at a pediatric age (2–11).

At present, the oldest age of PTC patients from the high-risk group is around 50, so currently, the focus of studies on Chernobyl thyroid cancer is shifting to middle-aged adults exposed to radioactive fallout in childhood and adolescence. Repeated screenings of members of the Ukrainian-American thyroid cohort have shown that 30 years after the Chernobyl accident, a statistically significant elevated risk of radiogenic thyroid cancer was still observed despite a gradual decrease over time from the first (1998–2000) to the fifth (2012–2015) rounds, i.e., with the longer period of latency (time between the Chernobyl accident and diagnosis of thyroid cancer) (12–14).

During 20 years after the Chernobyl accident, a high dose-dependent prevalence of driver gene fusions was reported in PTCs in children and adolescents (10) or young adults aged <30 years at surgery (15, 16). With latency period exceeding 20 years and patient age at surgery reaching 45, point mutations are becoming more frequent (17).

In view of the increasing role of point mutations, among which the *BRAF*^*V*600*E*^ is the most common (17), it seemed timely to evaluate its impact on potentially radiogenic PTCs in patients of advancing age. In non-irradiated adult patients, the *BRAF*^*V*600*E*^ mutation has been associated with a more aggressive tumor phenotype (18–25) and higher risk of recurrence [meta-analyses (19, 21, 23–26)], but no studies have been performed in individuals exposed to internal radiation. Therefore, we set out to address the BRAF^V600E^ relationships to various demographic and clinicopathological features, and environmental exposure in a large group of patients with Chernobyl PTC from Ukraine diagnosed during the 30 years after the accident.

## Materials and Methods

### Patients

A total of 428 patients aged from 4 to 49 years at the time of surgery who were operated on for PTC at the State Institution “V.P. Komisarenko Institute of Endocrinology and Metabolism of the National Academy of Medical Sciences of Ukraine” (IEM, Kyiv) from 1990 to 2017 were enrolled. All patients were ≤18 years of age at the time of the Chernobyl accident, lived in the six most ^131^I contaminated northern regions of Ukraine, and thus belonged to the high-risk group for the development of radiogenic thyroid cancer.

The study was conducted according to the guidelines of the Declaration of Helsinki and was approved by the IEM Bioethics Committee (protocols N 22-KE of 26 April 2018 and N 31-KE of 27 February 2020), the Chernobyl Tissue Bank (CTB, project N001-2020), and the Ethics Committee of Nagasaki University (protocol 20130401–7 of 1 July 2021, the latest update). Informed consent was obtained from all subjects involved in the study or their guardians (for minors).

### Histopathology

The primary analysis of histological specimens stained with hematoxylin/eosin, and PTC diagnosis was made in IEM by two experienced pathologists (TB and LZ). The pathological diagnosis was based on the 4th edition of the WHO histological classification (27). Most cases were reviewed by the international pathology panel of the Chernobyl tissue bank (CTB) project (28, 29). The diagnosis of PTC was confirmed in all cases. TNM categories were determined according to the 8th edition of the TNM classification (30).

PTCs were characterized by tumor size, dominant histological growth pattern, oncocytic changes, and major characteristics of tumor invasiveness (11, 31, 32). We also used an integrative clinicopathological variable, the “invasiveness score,” which is the arithmetic sum of every instance of multifocality, lymphatic/vascular invasion, any extrathyroidal extension (i.e., minimal or gross), N1 and M1, either isolated or in combination with other(s) for each tumor (32, 33). Thus defined, the invasiveness score ranged from 0 (no invasive feature presents) to 5 (all features present).

Clinical information was retrieved from the database of IEM. During the follow-up, patients received neck ultrasounds, and those thyroidectomized also received serum thyroglobulin tests on a 6-month to 1-year basis. Recurrence was defined as a tumor focus detected and treated not earlier than 6 months after the primary surgery. Radioiodine-refractory (RAI-R) recurrences were determined according to the guidelines of the American Thyroid Association (34).

### Immunohistochemistry

Immunohistochemical (IHC) staining for BRAF^V600E^ (LZ, TIR) was performed as described before (33). In brief, we used a mouse monoclonal anti-BRAF (mutated V600E) antibody (VE1) ab228461(Abcam) at a 1:100 dilution and the Novolink Polymer Detection System (250T) (Leica RE7140-K) to detect IHC reaction product. IHC staining was evaluated by three qualified observers (L.Z., T.B., T.I.R.), and full agreement was achieved; there were no specimens interpreted by any observer as potentially false-negative or false-positive. A close correlation between the results of the VE1-based IHC for BRAF^V600E^ and molecular methods of the detection of the *BRAF*^*V*600*E*^ mutation at the DNA level has been reported in a meta-analysis (35) and confirmed in our previous study using formalin-fixed paraffin-embedded material (36). Therefore, we assumed the BRAF^V600E^ positivity on IHC was indicative of the *BRAF*^*V*600*E*^ mutation.

The proliferative activity of tumors was evaluated by IHC using Ki67 antibody (clone MIB-1; DAKO, Glostrup, Denmark, 1:100 dilution) in a Ventana BenchMark ULTRA instrument. The Ki67 labeling index (Ki67 LI) was determined with the image-analyzing software (CountσCell, Ki67 antigen Semi-Auto Counter, Seiko Tec LTD, Fukuoka, Japan) in a total of ~1,000 PTC cells (LZ). Image analysis was performed in a blind for the BRAF^V600E^ status manner.

### Thyroid Dosimetry

The individual ^131^I thyroid radiation doses (the absorbed dose estimates in mGy) were calculated for each patient in the Dosimetry and Radiation Protection Department of the State Institution “National Research Center for Radiation Medicine of the National Academy of Medical Sciences of Ukraine” (NRCRM, Kyiv) using dosimetric models which include the system of ecological iodine transport and iodine biokinetics (“TD-CTB”) depending on the availability of direct measurements of thyroid activity in May-June 1986 in the person or in other persons in the same or adjacent settlements (37).

### Probability of Causation (POC) From Radiation

The probability of causation of a tumor by radiation exposure in a person of a given sex and age after a certain period of latency was determined using the US NIH/NCI Division of Cancer Epidemiology and Genetics' Interactive RadioEpidemiological Program—Probability of Cancer Causation from Radiation Version 5.7.1 software (38, 39). This software uses “Personal Information” such as gender, birth year, diagnosis year and cancer model (here, the “Thyroid (193)”), and “Dose Exposure Information” such as exposure year (here, 1986), exposure rate (here, the acute), radiation type (here, the electrons E > 15 keV as 90% of ^131^I beta-decay has the energy of 606 keV), organ dose (here, Constant) and parameter 1 (the thyroid dose in cSv; since radiation weighting factor for the beta-particles is 1, the equivalent doses were considered to be equal to the absorbed doses) as input variables. The output is the values of the “Assigned Share (Probability of Causation)” that range from the 1st to the 99^th^ percentile based on 10,000 random-seeded simulations. The higher POC value reflects the higher likelihood of cancer development due to radiation exposure. We used the median value (the 50th percentile) for calculations.

### Statistical Analysis

The Fisher's exact test, Fisher-Freeman-Halton exact test, and Cochran-Armitage test were used for univariate analysis of categorical data; the Mann-Whitney test was used to compare continuous data between two independent groups. Logistic regression models were adjusted for sex. Models with very small numbers of outcomes (<5 per cell) were conducted using Firth's approach to bias-reducing penalized maximum likelihood fit. Categorial variables with more than two response levels were assessed using multinomial logistic regression. Multivariate linear regression models were applied to continuous dependent variables. The BRAF^V600E^ effect in relation to the period of latency was estimated using survival analysis methods. The Kaplan-Meier method, the proportional hazard (Cox), and extended proportional hazard models were used. Computation and plotting of the results of the model with time-varying coefficients were performed with a SAS macro “coxtvc” (40). Multivariate models of the development of RAI-R recurrent PTC metastases in time were developed by non-automatic variable selection in the Cox proportional hazard model using minimization of the Akaike information criterion method. The integrated time-dependent area under curve was calculated for each model.

Additional adjustment for age at operation was performed to evaluate the impact of this variable as a confounder in separate models. Matching 1:1 the BRAF^V600E^-positive to the BRAF^V600E^-negative PTCs by age at operation (±2 years) was performed with the SAS macro “match” (https://git hub.com/Jiangtang/Programming-SAS/blob/master/UserMacros/ mayo/match.sas) using the “optimal” method. For analyses of matched groups, the univariate related samples tests, i.e., the Wilcoxon signed-rank test for continuous data, McNemar test for 2x2 contingency tables, and Bowker test for categorical variables with several response levels (SAS PROC FREQ with the “agree” option), and clustered log-rank test (41) for the period of latency and recurrence-free survival were applied. Multivariate analysis included linear regression for continuous data and conditional logistic regression analysis for categorical variables with a dichotomous response (SAS PROC LOGISTIC with the STRATA statement). The models included age at operation as an explanatory variable to control for residual confounding.

Calculations were performed using version 9.4 of SAS (SAS Institute, Cary, NC, USA) or IBM SPSS Statistics Version 24 software (International Business Machines Corp., Armonk, NY, USA). All tests were two-sided; p < 0.05 was considered statistically significant.

## Results

### Baseline and Radiation Exposure Characteristics

**Figure 1:**
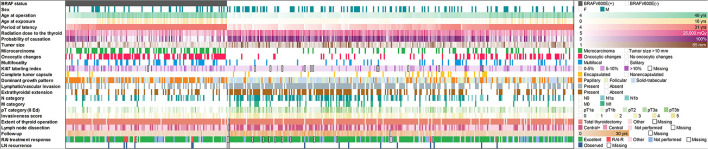
Baseline, radiation exposure, and clinicopathological profiles of 428 radiogenic PTCs in the study arranged by IHC BRAF status.

**Figure 2:**
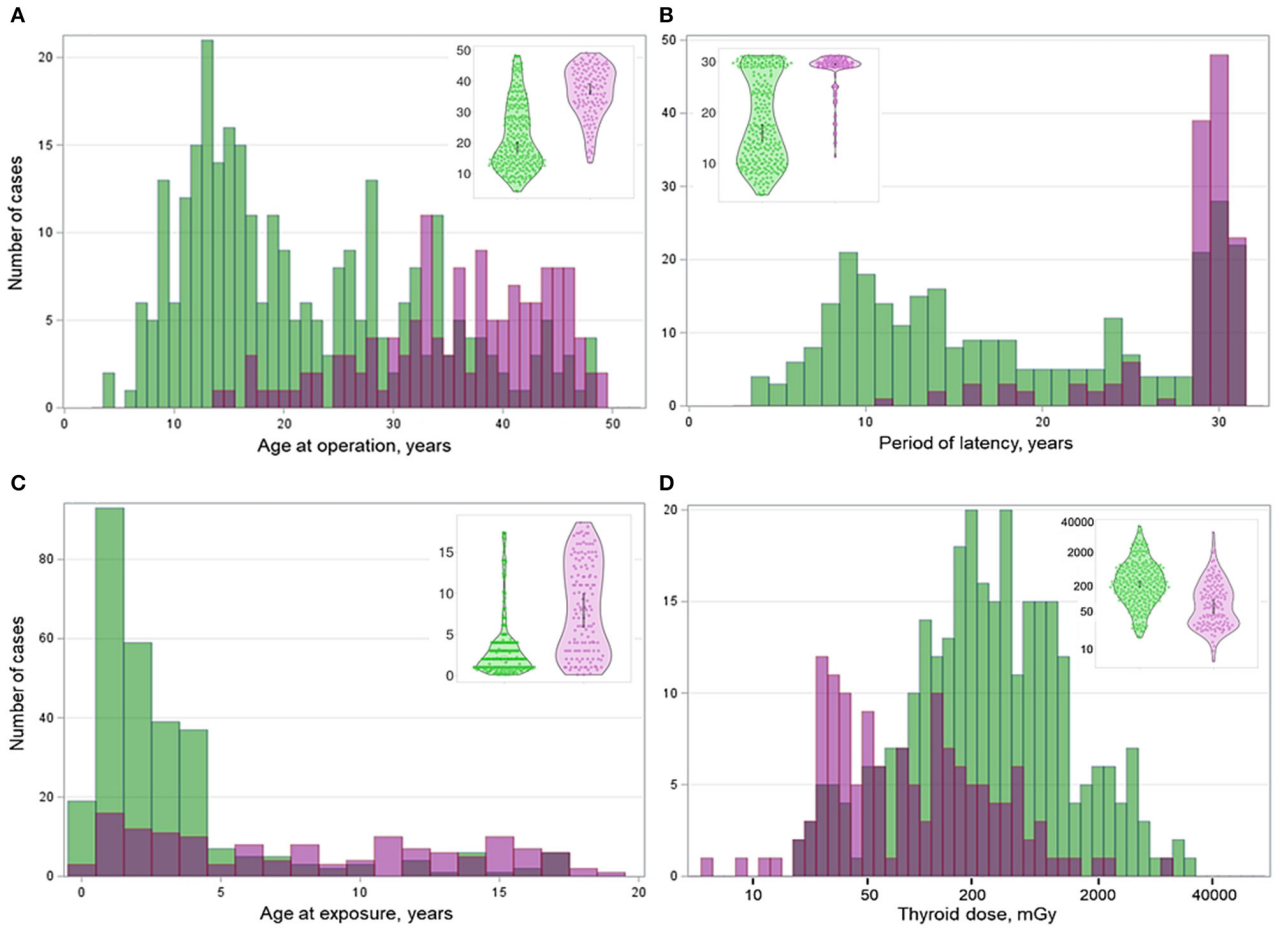
Distribution of the BRAF^V600E^-positive (purple color) and BRAF^V600E^-negative (green color) radiogenic PTCs by **(A)** age at operation, **(B)** age at exposure, **(C)** period of latency, and **(D)** thyroid dose. Insets are violin plots of corresponding distributions; the gray boxes inside indicate the 95% confidence intervals.

All data collected for or generated during this study are presented in Figure 1. Cytoplasmic expression of BRAF^V600E^ (indicative of the *BRAF*^*V*600*E*^ mutation at the DNA level) was observed in 136/428 (31.8%) of cases. As shown in Table 1, the proportion of male patients was lower in the BRAF^V600E^-positive PTCs (OR = 0.609, *p* = 0.037); therefore, the regression models were adjusted for sex. The distributions of the BRAF^V600E^-positive and BRAF^V600E^-negative PTCs by age at operation, age at exposure, latency, and ^131^I thyroid dose shown in Figure 2 strongly suggest substantial differences between the two PTC groups. Indeed, the BRAF^V600E^-positivity was associated with significantly older age at operation (b = 14.346, *p* < 0.001), age at exposure (b = 4.858, *p* < 0.001), and longer period of latency (b = 9.681, *p* < 0.001). The onset of PTCs with different BRAF status in time (i.e., after a certain latency) assessed in a Cox model did not meet the assumption of proportional hazards (Supplementary Figures 1A–C). We, therefore, used an extended Cox model with time-dependent BRAF status, which performed adequately (Supplementary Figure 1D). Parameters of the model confirmed the delay of the BRAF^V600E^-positive PTC development in time (HR = 0.007, *p* < 0.001) and their accelerated failure time behavior (HR = 1.181, *p* < 0.001 for the BRAF status^*^latency variable, Table 1).

**Table 1:** Comparative characteristics of the BRAF^V600E^-positive *versus* BRAFV^600E^-negative PTCs.

**Parameters**	**BRAF^V600E^(+) (*n* = 136)**	**BRAF^V600E^(-) (*n* = 292)**	***p*** **-value**	**OR, b, or HR (95% CI)^a^**	***p*** **-value**
	**Number or median** **(% or IQR)**	**Number or median** **(% or IQR)**	**Univariate**	**Multivariate logistic, linear, or proportional hazard regression**	
**Sex** F/M (%M; F:M ratio)	104/32 (23.5%; 3.3:1)	194/98 (33.6%; 2.0:1)	**0.042**	**0.609 (0.383–0.969)** ^b^	**0.037** ^c^
**Age at operation**, years	37.8 (32.0–43.2)	18.7 (13.1–28.7)	**<0.001**	**14.346 (12.293–16.400)**	**<0.001**
**Age at exposure**, years	7.8 (3.0–13.3)	2.0 (1.0–4.0)	**<0.001**	**4.858 (3.959–5.757)**	**<0.001**
**Period of latency**, years	29.5 (28.8–30.2)	16.3 (10.4–27.7)	**<0.001**	**9.681 (8.165–11.197)**	**<0.001**
Period of latency^d^				**0.007 (0.002–0.026)** ^d^	**<0.001**
BRAF status*latency^d^				**1.181 (1.128–1.236)** ^d^	**<0.001**
**Radiation dose to the thyroid**, mGy	68 (29–180)	254 (106–673)	**<0.001**	**−560.333 (−959.536 - −161.130)**	**0.006** ^c^
**Probability of causation (POC)**, %	23.0 (7.5–55.9)	64.9 (41.9–83.7)	**<0.001**	**−27.534 (−33.126 -** **−21.941)**^b^	**<0.001**
≤25%	69 (50.7%)	38 (13.0%)	**<0.001**	**6.884 (4.265–11.111)** ^b^	**<0.001**
>25–50%	32 (23.5%)	75 (25.7%)	0.719	0.890 (0.553–1.432)^b^	0.632
>50–75%	22 (16.2%)	85 (29.1%)	**<0.001**	**0.470 (0.279–0.792)** ^b^	**0.005**
>75–100%	13 (9.6%)	94 (32.2%)	**<0.001**	**0.223 (0.120–0.415)** ^b^	**<0.001**
**Tumor size**, mm	11 (7–18)	15 (12–27)	**<0.001**	**−7.119 (−9.648 - −4.591)**	**<0.001**
≤10 mm (microcarcinoma)	68 (50.0%)	50 (17.1%)	**<0.001**	**4.788 (3.036–7.552)**	**<0.001**
11–20 mm	47 (34.6%)	144 (49.3%)	**0.005**	**0.544 (0.356–0.831)**	**0.005**
21–40 mm	19 (14.0%)	68 (23.3%)	**0.028**	**0.548 (0.314–0.958)**	**0.034** ^c^
40+ mm	2 (1.5%)	30 (10.3%)	**<0.001**	**0.125 (0.029–0.532)**	**0.005** ^c^
**Oncocytic changes**	78 (57.4%)	62 (21.2%)	**<0.001**	**4.820 (3.091–7.515)**	**<0.001**
**Multifocality**	43 (31.6%)	44 (15.1%)	**<0.001**	**2.539 (1.562–4.127)**	**<0.001** ^c^
**Ki67 labeling index**	*n* = 135; 4.6 (3.6–6.3)	*n* = 281; 2.7 (1.6–4.6)	**<0.001**	**1.747 (1.148–2.346)**	**<0.001**
Ki67 group			**<0.001**	**2.441 (1.573–3.787)** ^f^	**<0.001**
≤5%	78 (57.8%)	219 (77.9%)	**<0.001**	**0.393 (0.252–0.613)**	**<0.001**
>5–10%	48 (35.6%)	50 (17.8%)	**<0.001**	**2.553 (1.597–4.080)**	**<0.001**
>10%	9 (6.7%)	12 (4.3%)	0.34	1.500 (0.613–3.669)	0.375
**Complete tumor capsule**	5 (3.7%)	32 (11.0%)	**0.015**	**0.297 (0.113–0.783)**	**0.014** ^c^
**Dominant growth pattern**			**<0.001**	**0.316 (0.213–0.467)** ^f^	**<0.001**
Papillary	76 (55.9%)	61 (20.9%)	**<0.001**	**4.715 (3.029–7.338)**	**<0.001**
Follicular	17 (12.5%)	97 (33.2%)	**<0.001**	**0.302 (0.171–0.532)**	**<0.001**
Solid–trabecular	43 (31.6%)	134 (45.9%)	**0.006**	**0.523 (0.339–0.806)**	**0.003** ^c^
**Lymphatic/vascular invasion**	48 (35.3%)	190 (65.1%)	**<0.001**	**0.303 (0.198–0.466)**	**<0.001** ^c^
**Extrathyroidal extension (any)**	37 (27.2%)	140 (47.9%)	**<0.001**	**0.417 (0.267–0.650)**	**<0.001** ^c^
**N category (N1)**	45 (33.1%)	145 (49.7%)	**0.002**	**0.518 (0.338–0.793)**	**0.003** ^c^
N1a	23 (16.9%)	51 (17.5%)	1.000	0.965 (0.561–1.662)	0.899
N1b	22 (16.2%)	94 (32.2%)	**<0.001**	**0.422 (0.250–0.710)**	**0.001** ^c^
**M category (M1)**	2 (1.5%)	41 (14.0%)	**<0.001**	**0.091 (0.022–0.383)**	**0.001** ^c^
**pT category (8 Ed)**			**<0.001**	**0.307 (0.185–0.511)** ^f^	**<0.001**
pT1	113 (83.1%)	179 (61.3%)	**<0.001**	**3.075 (1.850–5.112)**	**<0.001**
pT1a	68 (50.0%)	50 (17.1%)	**<0.001**	**4.816 (3.053–7.598)**	**<0.001**
pT1b	45 (33.1%)	129 (44.2%)	**0.034**	**0.622 (0.406–0.954)**	**0.030**
pT2	17 (12.5%)	52 (17.8%)	0.204	0.673 (0.372–1.218)	0.191
pT3	6 (4.4%)	61 (20.9%)	**<0.001**	**0.173 (0.073–0.413)**	**<0.001** ^c^
pT3a	2 (1.5%)	19 (6.5%)	**0.029**	**0.210 (0.048–0.919)**	**0.038** ^c^
pT3b	4 (2.9%)	42 (14.4%)	**<0.001**	**0.180 (0.063–0.515)**	**0.001** ^c^
**Invasiveness score (any Ex)**	1 (0–2)	2 (1–3)	**<0.001** ^e^	**0.485 (0.335–0.702)** ^f^	**<0.001** ^c^
0	48 (35.3%)	63 (21.6%)	**0.003**	**1.977 (1.259–3.105)**	**0.003** ^c^
1	32 (23.5%)	59 (20.2%)	0.448	1.173 (0.718–1.918)	0.524
2	29 (21.3%)	68 (23.3%)	0.711	0.855 (0.521–1.404)	0.536
3	23 (16.9%)	58 (19.9%)	0.510	0.889 (0.518–1.527)	0.670
4	4 (2.9%)	32 (11.0%)	**0.005**	**0.247 (0.085–0.716)**	**0.010** ^c^
5	0	12 (4.1%)	**0.012**	0.085 (0.005–1.404)	0.085
**Extent of thyroid operation**		*n* = 291	**0.033**		**0.047**
Total thyroidectomy	131 (96.3%)	263 (90.4%)		**2.690 (1.012–7.153)**	
Other	5 (3.7%)	28 (9.6%)		**0.372 (0.140–0.988)**	
**Lymph node dissection performed**	66 (48.5%)	n=291; 162 (55.7%)	0.177	0.777 (0.515–1.173)	0.230
Level ≥6	41 (30.1%)	55 (18.9%)	**<0.001**	**2.487 (1.512–4.091)**	**<0.001** ^c^
Level 1-−5	25 (18.4%)	107 (36.8%)	**<0.001**	**0.402 (0.244–0.661)**	**<0.001** ^c^
**RAI treatment performed**	*n* = 135; 112 (83.0%)	*n* = 279; 227 (81.4%)	0.786	1.111 (0.646–1.912)	0.704
**Follow–up, yrs**	3.7 (2.3–5.1)	*n* = 290; 9.7 (3.6–16.9)	3.22E−14	**−6.129 (−7.498 - −4.760)**	**<0.001** ^c^
**LN recurrences (reoperated after 6 mo)**	6 (4.4%)	*n* = 290; 9 (3.1%)	0.574	1.421 (0.493–4.099)	0.516
**Recurrence–free survival**		*n* = 290	**0.003** ^g^	**5.075 (1.515–17.007)** ^h^	**0.008** ^c^
**RAI treatment response**	*n* = 112	*n* = 227	**0.072**	**0.492 (0.249–0.971)** ^f^	**0.041** ^c^
RAI–R recurrence vs. other	6 (5.4%)	2 (0.9%)	**0.018**	**7.113 (1.378–36.717)**	**0.019** ^c^
excellent vs. other	94 (83.9%)	206 (90.7%)	0.072	0.520 (0.262–1.030)	0.061

a *Adjusted for sex unless otherwise specified*.

b *Non-adjusted*.

c *Statistical significance was lost after the additional adjustment for age at operation (see Supplementary Table 4)*.

d *The extended proportional hazard regression*.

e *The trend test (the Cochran-Armitage test)*.

f *Multinomial logistic regression*.

g *The log-rank test*.

h *The proportional hazard model*.

*Numbers in bold indicate statistical significance*.

The BRAF^V600E^-positivity was associated with lower ^131^I thyroid dose (b = −560.333, *p* = 0.006) and POC (b = −27.534, *p* < 0.001). With increasing patient age at exposure, POC was decreased in all dose ranges, both for the BRAF^V600E^-positive and -negative PTCs (Supplementary Figure 2).

### Histopathological Characteristics

The BRAF^V600E^-positive PTCs were characterized by a smaller size (b = −7.119, *p* < 0.001) and higher frequencies of microcarcinomas (OR = 4.788, *p* < 0.001). Among microcarcinomas, there were no BRAF^V600E^-positive and only 2 BRAF^V600E^-negative incidentalomas. The higher frequencies were observed for oncocytic changes in tumor cells (OR = 4.820, *p* < 0.001), multifocal growth (OR = 2.539, *p* < 0.001), higher Ki67 LI (b = 1.747, *p* < 0.001) due to more frequent Ki67 LI from 5 to 10% (OR = 2.553, *p* < 0.001), while the frequency of fully encapsulated PTCs was lower (OR = 0.297, *p* = 0.014) (Table 1). Of interest, the size of the BRAF^V600E^-positive PTCs did not correlate with Ki67 LI (*b* = −0.008, *p* = 0.938) while there was a significant inverse correlation in the BRAF^V600E^-negative group (*b* = −0.127, *p* = 0.027); the difference in these regression coefficients was statistically significant (*p*_het_ = < 0.001).

With regard to tumor architecture, the dominant papillary growth pattern was most common for the BRAF^V600E^-positive PTCs (OR = 4.715, *p* < 0.001). Follicular and solid-trabecular dominant components were less frequent (OR = 0.302, *p* < 0.001; and OR = 0.523, *p* = 0.003, respectively). Note that the dominant structural component generally coincides with histological PTC subtype/variant or implies conventional PTC if a tumor has a mixed structure (Supplementary Table 1).

**Figure 3:**
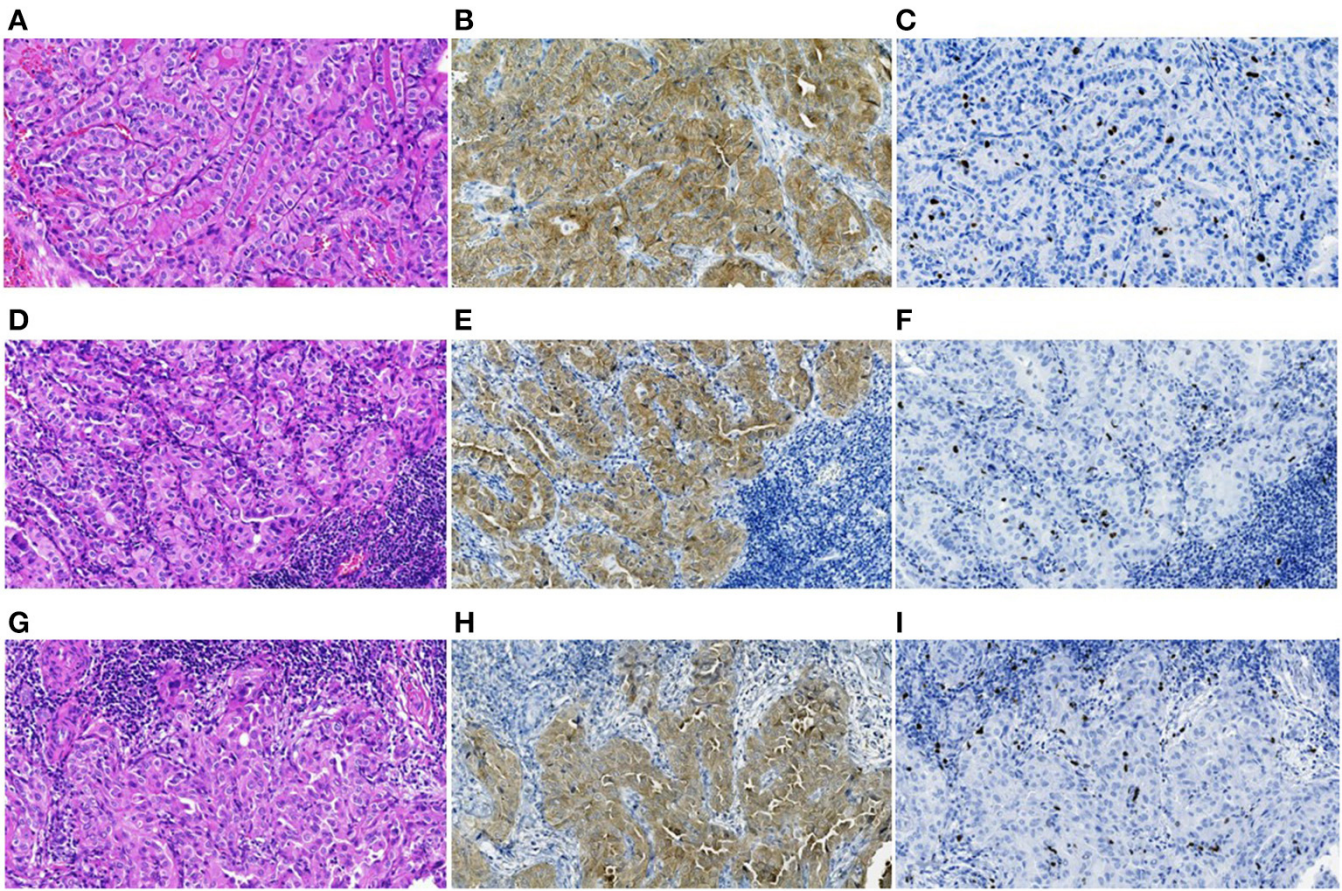
The BRAF^V600E^-positive radiogenic PTC: **(A–C)** primary tumor, **(D–F)** primary lymph node metastasis, and **(G–I)** an RAI-R recurrent metastasis removed 2.8 years after the first surgery, all images are at 200X magnification. **(A)** Primary tumor with trabecular-papillary growth pattern, tall cell variant, H&E; **(B)** positive IHC reaction with anti-BRAF (mutated V600E) antibody; **(C)** IHC reaction with Ki67 (Clone MIB-1) antibody (Ki67 LI 5.4%). **(D)** Primary oncocytic cell metastasis with solid-trabecular dominant growth pattern, H&E; **(E)** positive IHC reaction with anti-BRAF antibody; **(F)** IHC reaction with Ki67 antibody (Ki67 LI 3.3%). **(G)** RAI-R recurrent oncocytic cell metastasis with solid-trabecular growth pattern, H&E; **(H)** positive IHC reaction with anti-BRAF antibody; **(I)** IHC reaction with Ki67 antibody (Ki67 LI 3.3%).

Only 17/136 (12.5%) of the BRAF^V600E^-positive PTCs belonged to rare histological variants, of which 9 (6.6%) were the tall cell and 8 (5.9%) were the Warthin-like variants (Supplementary Table 1). These tumors were characterized by pronounced oncocytic changes not only in the cells of the primary tumor but also in the cells of lymph node metastases (Figure 3). Among the BRAF^V600E^-negative PTCs, rare histological variants were even less common 13/292 (4.4%) of cases (*p* = 0.004 for frequency comparison with the BRAF^V600E^-positive PTCs). The most frequent was the diffuse sclerosing variant, 11/292 (3.8%) of cases; the tall cell and hobnail variants were represented by one case (0.3%) each. The distribution of particular rare histological variants in the BRAF^V600E^-positive and BRAF^V600E^-negative PTCs was statistically significantly different (*p* < 0.001).

The BRAF^V600E^-positive PTCs displayed less frequent invasive features: lymphatic/vascular invasion (OR = 0.303, *p* < 0.001), extrathyroidal extension (OR = 0.417, *p* < 0.001), regional (OR = 0.518, *p* = 0.003) and distant metastases (OR = 0.091, *p* = 0.001), and the integrative invasiveness score (OR = 0.485, *p* < 0.001; Table 1). To evaluate the impact of rare histological variants on tumor invasiveness, we compared PTCs of common variants only (i.e., without rare variants). The effects of BRAF^V600E^ were very similar to the results presented in Table 1: less frequent lymphatic/vascular invasion (OR = 0.308, *p* < 0.001), extrathyroidal extension (OR = 0.425, *p* < 0.001), regional (OR = 0.514, *p* = 0.004) and distant metastases (OR = 0.117, *p* = 0.004), and the integrative invasiveness score (OR = 0.495, *p* < 0.001). Hence, rare histological variants did not markedly contribute to the differences in tumors invasiveness observed between the BRAF^V600E^-positive and BRAF^V600E^-negative PTCs.

Within the BRAF^V600E^-positive group, we did not find signs of higher aggressiveness of PTCs of rare variants as compared to those of PTCs of common variants (the strongest OR = 1.505, *p* = 0.442; all 95% CIs for ORs included the value of 1). In contrast, in the BRAF^V600E^-negative group, PTCs of rare variants had distant metastases more frequently (OR = 4.517, *p* = 0.014) and higher invasiveness score (OR = 2.867, *p* = 0.039); associations for other invasive features were statistically non-significant, although all ORs were >1 (data not shown).

### Clinical Characteristics

Total thyroidectomy was the principal extent of operation in more than 90% of cases in both BRAF^V600E^-positive and BRAF^V600E^-negative groups (Table 1), although statistically, it was more frequent in the former (OR = 2.690, *p* = 0.047). Lymph node dissection was performed in about half of the cases without an overall difference between the groups (OR = 0.777, *p* = 0.230), but the central dissection was more frequent (OR = 2.487, *p* < 0.001) and the lateral less frequent in the BRAF^V600E^-positive group (OR = 0.402, *p* < 0.001). Most patients (>80%) in each group received postoperative RAI treatment (OR = 1.111, *p* = 0.704).

**Figure 4:**
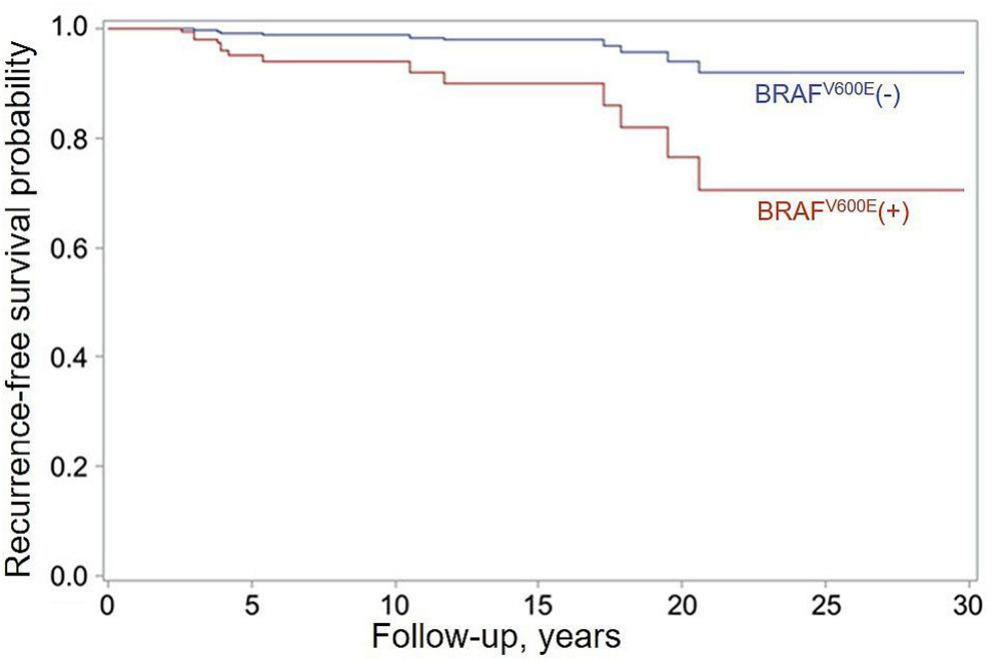
Recurrence-free survival function estimates for patients with the BRAF^V600E^-positive (red) and BRAF^V600E^-negative (blue) PTCs obtained in the Firth's-penalized Cox proportional hazard model adjusted for sex, tumor size, N and M categories, extrathyroidal extension, multifocality, lymphatic/vascular invasion, extent of thyroid operation, lymph node dissection, and RAI treatment. HR = 5.334 (95% CI, 1.196–24.746, *p* = 0.034) for the BRAF^V600E^-positive status.

The median follow-up of 3.7 years in the BRAF^V600E^-positive group was shorter than that of 9.7 years in the BRAF^V600E^-negative group (b = −6.129, *p* < 0.001), likely due to the longer period of latency of the BRAF^V600E^-positive tumors. In the course of follow-up, 6/136 (4.4%) of the BRAF^V600E^-positive PTCs and 9/290 (3.1%) of the BRAF^V600E^-negative PTCs (OR = 1.421, *p* = 0.516) developed recurrent lymph node metastases not earlier than 6 months after the primary surgery. Lymph node metastases were the only type of recurrences observed in this study, and all of them were reoperated. We confirmed the fully concordant BRAF^V600E^ status of the primary and recurrent tumors in all 15 recurrent cases (Cohen's κ = 1.000, *p* = 0.001). Analysis of disease-free survival in the Cox model showed that despite the relatively small absolute difference in the frequency of recurrences, the BRAF^V600E^ positivity was a risk factor for recurrence (HR = 5.334, *p* = 0.034 adjusted for sex, tumor size, N and M categories, extrathyroidal extension, multifocality, lymphatic/vascular invasion, extent of thyroid operation, lymph node dissection, and RAI treatment, which are the factors potentially affecting the chance of recurrence; Figure 4). The effect of BRAF^V600E^ was nearly independent of other variables as judged from the HR = 5.075 value after adjustment for sex only (see Table 1).

Comparative characteristics of the primary tumors and recurrent metastases with different BRAF statuses are presented in more detail in Supplementary Table 2. No difference in tumor size was observed, yet of note, among all recurrent PTCs there was only one BRAF^V600E^-negative and zero BRAF^V600E^-positive microcarcinomas, and all primary tumors were non-encapsulated. The recurrent BRAF^V600E^-positive primary tumors displayed oncocytic changes and multifocality more frequently than the BRAF^V600E^-negative PTCs.

It is unlikely that rare PTCs or those associated with higher tumor aggressiveness histological subtypes were overrepresented among the recurrent PTCs. There were only one (of a total of eight, 12.5%) Warthin-like and one (of a total of nine, 11.1%) tall cell variants among the BRAF^V600E^-positive PTCs and two (of a total 11, 18.2%) diffuse-sclerosing variants among the BRAF^V600E^-negative primary PTCs that recurred.

Of importance, all 6 (100%) recurrent BRAF^V600E^-positive metastases were radioiodine-refractory (RAI-R) while there were only 2/9 (22.2%) of such among the BRAF^V600E^-negative recurrences (Table 1 and Figure 3). The difference in frequencies of the RAI-R recurrent metastases was statistically significant (OR = 7.113, *p* = 0.019). In view of clinical significance of RAI-R recurrences, we created several multivariate models (Table 2). Histopathological characteristics associated with RAI-R in these models were greater tumor size, oncocytic changes, multifocality, and the BRAF^V600E^-positivity. As seen in Table 1, most of these variables are closely related to BRAF^V600E^.

**Table 2:** Multivariate proportional hazard models of RAI-R recurrence.

**Parameters**	**HR (95% CI)**	***p*** **-value**	**AIC^a^**	**AUC^b^**
**Model 1**			59.497	0.947
Tumor size, mm	1.069 (1.009–1.131)	0.022		
Multifocality	10.907 (1.561–76.196)	0.016		
Dominant papillary growth pattern	7.326 (0.794–67.615)	0.079		
Oncocytic changes	25.204 (3.377–267.256)	0.007		
**Model 2**			64.407	0.884
BRAF^V600E^(+)	2.950 (0.548–24.758)	0.249		
Multifocality	6.400 (1.293–47.653)	0.035		
Oncocytic changes	12.074 (1.740–247.538)	0.031		
**Model 3**			66.178	0.872
BRAF^V600E^(+)	21.725 (3.183–266.311)	0.005		
Tumor size, mm	1.060 (1.008–1.117)	0.022		
Multifocality	6.916 (1.533–48.440)	0.021		

a *Akaike information criterion*.

b *Integrated time-dependent area under the curve*.

No deaths were documented in the group of 136 patients with the BRAF^V600E^-positive PTCs, and 4 deaths were registered among 292 patients (1.4%) with BRAF^V600E^-negative PTCs (*p* = 0.312) from 9 to 25 years after surgery, but none was due to PTC progression.

### Patient Age as a Confounder

During exploratory data analysis, we noticed that age at operation was strongly correlated with the BRAF status (which was the focus of this study, Table 1) and a number of baseline or tumor characteristics (which were considered the outcomes; Supplementary Table 3). Correlations of age at operation with the outcomes, however, could not be construed as causative, indicating that this variable may be a confounder. To account for its effect, we used two statistical solutions.

First, we adjusted the multivariate regression models for age at operation. The BRAF^V600E^ positivity retained significant associations with older age at exposure, a longer period of latency, lower POC, smaller tumor size, higher frequency of oncocytic changes, higher KI67 LI, higher frequency of dominant papillary growth pattern, and lower frequency of the follicular pattern (Supplementary Table 4). No statistically significant associations with aggressive features were seen. Among clinical characteristics, only a higher frequency of total thyroidectomy was observed. The associations were lost for radiation dose to the thyroid, large-size tumors, multifocality, tumor capsule, lymphatic/vascular invasion, extrathyroidal extension, regional and distant metastasis, and the integrative invasiveness score (see also Table 1 footnote “c”). No associations were longer seen with lymph node dissections, recurrence-free survival, and RAI treatment response.

Second, we performed fuzzy (± 2 years) 1:1 BRAF^V600E^-positive-BRAF^V600E^-negative PTCs matching by age at operation. On multivariate analysis, the association with smaller tumor size (and microcarcinomas), papillary growth pattern, and higher Ki67 LI remained statistically significant (Supplementary Table 5), implying these are inherent properties of the BRAF^V600E^-positive radiogenic PTCs. The number of lost associations was even greater, including those with baseline characteristics, thyroid radiation dose and POC, aggressive tumor features, and clinical data.

The associations between BRAF^V600E^ and different characteristics that were lost after the varying-stringency adjustments for age at operation should be interpreted with caution since a potent confounder, when introduced in a multivariate statistical model, may mask/obscure the effects of causative explanatory variables. We, therefore, believe that the data presented in Table 1 (i.e., without adjustment for age at operation) describe the BRAF^V600E^ correlations with baseline and clinicopathological characteristics of radiogenic PTCs adequately.

### Relationship of the BRAF^V600E^ Status With the POC Level

**Figure 5:**
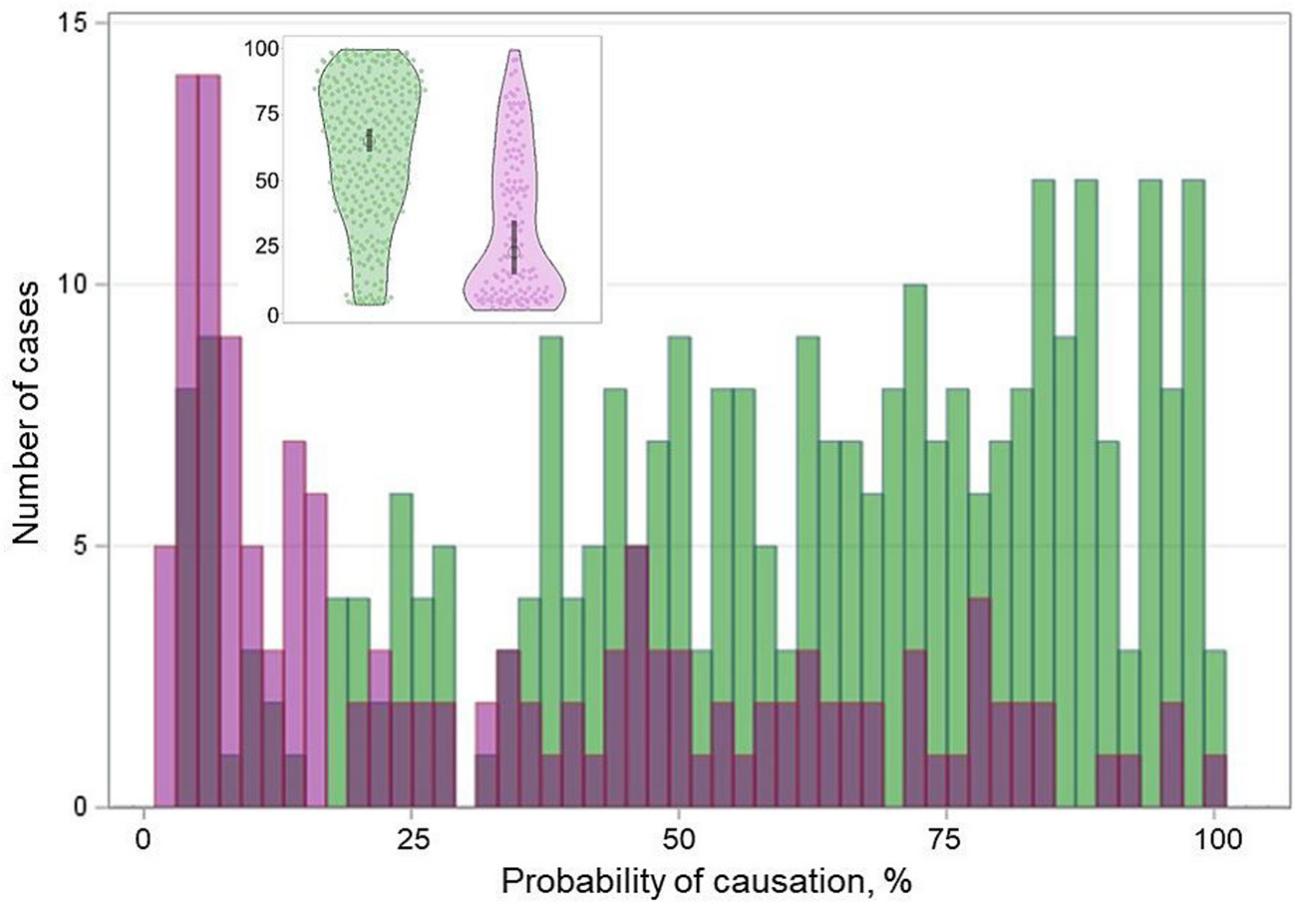
Distribution of the BRAF^V600E^-positive (purple color) and BRAF^V600E^-negative (green color) radiogenic PTCs by the probability of causation (POC). Inset is a violin plot of corresponding distribution; the gray boxes indicate the 95% confidence intervals.

The BRAF^V600E^-positive PTCs and BRAF^V600E^-negative PTCs displayed statistically significant differences in the probability of causation (POC) of a tumor due to radiation exposure (Table 1 and Figure 5). We therefore further examined whether comparative characteristics of tumors with different BRAF statuses were changing with regard to the POC level. For this purpose, we calculated the BRAF^V600E^ effect on PTC characteristics for different POC quartiles and then determined whether the linear trend for changes across POC quartiles was statistically significant.

#### Baseline Characteristics and Thyroid Radiation Dose

The frequencies of patient sex did not display differences between the BRAF^V600E^-positive PTCs and BRAF^V600E^-negative PTCs at any POC quartile and no significant trend was seen (*p*_trend_ = 0.276, Table 3). Age at operation of patients with BRAF^V600E^-positive tumors was significantly older in all POC quartiles, but the uptrend was only suggestive (*p*_trend_ = 0.081). Patient age at the time of exposure was also older in patients with the BRAF^V600E^-positive PTC in each quartile, with statistically significant differences in POC Q1 and Q2; the trend was statistically significantly descending (*p*_trend_ = 0.030). The longer latency of the BRAF^V600E^-positive PTCs was consistently observed in all POC quartiles, and there was a borderline significant uptrend (*p*_trend_ = 0.055) suggestive of a somewhat longer latency of the BRAF^V600E^-positive tumors with increasing POC.

**Table 3:** Comparison of the BRAF^V600E^-positive vs. BRAF^V600E^-negative PTCs by POC quartiles.

**POC quartiles (*n* = 107 each)**	**Q1 (1.3–24.5%, range)**		**Q2 (24.5–54.3%, range)**		**Q3 (54.3–78.8%, range)**		**Q4 (78.9–99.5%, range)**			
**Parameters**	**OR, HR, or b (95% CI)^a^**	***p*** **-value**	**OR, HR, or b (95% CI)^a^**	***p*** **-value**	**OR, HR, or b (95% CI)^a^**	***p*** **-value**	**OR, HR, or b (95% CI)^a^**	***p*** **-value**	***p***_trend_	**Trend direction**
	**Multivariate logistic, linear, or proportional hazard regression**		**Multivariate logistic, linear, or proportional hazard regression**		**Multivariate logistic, linear, or proportional hazard regression**		**Multivariate logistic, linear, or proportional hazard regression**			
**Sex**	0.625 (0.250–1.559)	0.313	0.444 (0.170–1.158)	0.097	0.429 (0.133–1.386)	0.157	2.615 (0.808–8.467)	0.109	0.276	+
**Age at operation**, years	**1.256 (0.572–1.940)**	**<0.001**	**1.989 (1.042–2.937)**	**<0.001**	**2.411 (1.516–3.306)**	**<0.001**	**2.403 (1.383–3.422)**	**<0.001**	0.081	+
**Age at exposure**, years	**0.490 (0.227–0.752)**	**<0.001**	**0.447 (0.027–0.867)**	**0.037**	0.254 (−0.164 - 0.671)	0.231	0.056 (−0.432 - 0.543)	0.821	**0.030**	–
**Period of latency**, years	**1.412 (0.562–2.262)**	**0.001**	**2.933 (1.469–4.397)**	**<0.001**	**4.136 (2.615–5.656)**	**<0.001**	**4.212 (2.409–6.015)**	**<0.001**	0.055	+
**Radiation dose to the thyroid**, mGy	−0.112 (0.244 - 0.019)	0.092	0.016 (−0.092 - 0.124)	0.772	0.0004 (−0.073 - 0.074)	0.991	−0.161 (−0.382 - 0.060)	0.152	0.756	–
**POC**, %	**−3.029 (−5.556 - −0.503)**	**0.019**	−1.200 (−4.817 - 2.418)	0.512	−0.705 (−3.977 - 2.566)	0.670	−3.651 (−7.380 - 0.078)	0.055	0.875	–
**Tumor size**, mm	**−0.428 (−0.796 - −0.060)**	**0.023**	**−0.763 (−1.162 - −0.364)**	**<0.001**	**−1.040 (−1.498 - −0.582)**	**<0.001**	−0.323 (−0.880 - 0.234)	0.253	0.985	+
≤ 10 mm (microcarcinoma)	**2.854 (1.172–6.952)**	**0.021**	**7.180 (2.637–19.551)**	**<0.001**	**13.561 (4.450–41.324)**	**<0.001**	1.428 (0.387–5.271)	0.593	0.950	+
11–20 mm	0.583 (0.257–1.326)	0.198	0.712 (0.297–1.709)	0.447	**0.299 (0.100–0.890)**	**0.030**	1.490 (0.440–5.045)	0.522	0.415	+
21–40 mm	1.039 (0.373–2.890)	0.942	**0.251 (0.079–0.801)**	**0.019**	0.133 (0.017–1.054)	0.056	0.513 (0.059–4.486)	0.546	0.456	–
40+ mm	**0.048 (0.003–0.862)**	**0.039**	0.316 (0.037–2.719)	0.294	0.462 (0.054–3.955)	0.481	0.310 (0.016–5.873)	0.435	0.302	+
**Oncocytic changes**	2.195 (0.973–4.952)	0.058	**4.668 (1.847–11.800)**	**0.001**	**4.920 (1.730–13.993)**	**0.003**	**4.804 (1.367–16.875)**	**0.014**	0.201	+
**Multifocality**	2.254 (0.809–6.275)	0.120	**6.008 (2.071–17.429)**	**0.001**	2.091 (0.708–6.180)	0.182	1.265 (0.307–5.206)	0.745	0.580	–
**Ki67 labeling index**	**0.366 (0.044–0.688)**	**0.026**	**0.757 (0.361–1.153)**	**<0.001**	**0.729 (0.272–1.186)**	**0.002**	**1.016 (0.411–1.621)**	**0.001**	0.072	+
Ki67 group	1.200 (0.515–2.797)	0.672	**2.981 (1.240–7.165)**	**0.015**	**2.817 (1.046–7.587)**	**0.040**	**4.437 (1.332–14.778)**	**0.015**	0.069	+
0–5%	0.801 (0.340–1.889)	0.613	**0.304 (0.124–0.745)**	**0.009**	0.370 (0.135–1.018)	0.054	**0.199 (0.058–0.687)**	**0.011**	0.151	–
>5–10%	1.303 (0.532–3.189)	0.563	**3.283 (1.273–8.468)**	**0.014**	2.170 (0.765–6.153)	0.145	**4.684 (1.309–16.770)**	**0.018**	0.202	+
>10%	0.897 (0.141–5.721)	0.909	1.573 (0.326–7.600)	0.573	3.313 (0.432–25.428)	0.249	2.850 (0.265–30.620)	0.387	0.122	+
**Complete tumor capsule**	0.481 (0.029–8.005)	0.610	0.079 (0.004–1.397)	0.083	0.431 (0.090–2.070)	0.293	3.602 (0.591–21.934)	0.164	0.238	+
**Dominant growth pattern**	**0.355 (0.164–0.765)**	**0.008**	0.536 (0.245–1.174)	0.119	**0.248 (0.099–0.620)**	**0.003**	**0.143 (0.043–0.481)**	**0.002**	0.289	–
Papillary	**3.655 (1.532–8.717)**	**0.003**	**2.865 (1.191–6.889)**	**0.019**	**5.194 (1.876–14.380)**	**0.002**	**11.063 (2.921–41.901)**	**<0.001**	0.145	+
Follicular	0.444 (0.160–1.229)	0.118	**0.257 (0.070–0.943)**	**0.040**	0.469 (0.142–1.552)	0.215	**0.114 (0.014–0.942)**	**0.044**	0.400	–
Solid–trabecular	0.457 (0.199–1.050)	0.065	0.825 (0.353–1.932)	0.658	0.386 (0.135–1.099)	0.075	0.409 (0.104–1.608)	0.201	0.635	–
**Lymphatic/vascular invasion**	0.621 (0.275–1.406)	0.253	**0.281 (0.114–0.691)**	**0.006**	**0.234 (0.085–0.646)**	**0.005**	0.520 (0.158–1.715)	0.283	0.758	–
**Extrathyroidal extension (any)**	0.884 (0.351–2.223)	0.793	0.768 (0.328–1.797)	0.543	**0.309 (0.101–0.940)**	**0.039**	0.293 (0.075–1.151)	0.079	0.061	–
**N category (N1)**	0.992 (0.428–2.298)	0.986	0.679 (0.290–1.590)	0.373	0.388 (0.128–1.181)	0.095	**0.149 (0.031–0.724)**	**0.018**	**0.002**	–
N1a	1.306 (0.418–4.077)	0.646	1.223 (0.435–3.441)	0.703	0.876 (0.224–3.428)	0.849	0.330 (0.040–2.754)	0.306	**0.047**	–
N1b	0.795 (0.292–2.168)	0.655	0.499 (0/178–1.394)	0.185	0.324 (0.084–1.254)	0.103	0.157 (0.019–1.275)	0.083	**0.011**	–
**M category (M1)**	0.170 (0.009–3.310)	0.242	0.104 (0.006–1.840)	0.122	0.416 (0.088–1.975)	0.270	0.212 (0.011–4.010)	0.301	0.580	+
**pT category (8 Ed)**	0.560 (0.225–1.392)	0.212	**0.217 (0.076–0.616)**	**0.004**	**0.201 (0.054–0.743)**	**0.016**	0.192 (0.023–1.637)	0.131	0.191	–
pT1	1.645 (0.655–4.132)	0.289	**4.987 (1.717–14.484)**	**0.003**	**4.794 (1.309–17.553)**	**0.018**	5.045 (0.619–41.111)	0.131	0.218	+
pT1a	**2.854 (1.172–6.952)**	**0.021**	**7.180 (2.637–19.551)**	**<0.001**	**10.999 (3.706–32.641)**	**<0.001**	1.988 (0.569–6.952)	0.282	0.962	+
pT1b	0.547 (0.240–1.248)	0.152	0.790 (0.328–1.901)	0.599	0.398 (0.133–1.189)	0.099	1.376 (0.419–4.524)	0.599	0.372	+
pT2	0.948 (0.337–2.670)	0.920	**0.182 (0.039–0.840)**	**0.029**	0.414 (0.087–1.973)	0.268	0.582 (0.064–5.254)	0.630	0.653	–
pT3	0.217 (0.037–1.269)	0.090	0.384 (0.102–1.443)	0.157	0.153 (0.019–1.220)	0.076	0.170 (0.009–3.263)	0.240	0.545	–
pT3a	**0.120 (0.000–0.808)**	**0.033**	0.701 (0.072–6.799)	0.759	0.886 (0.092–8.491)	0.916	0.399 (0.021–7.601)	0.541	0.609	+
pT3b	0.981 (0.085–11.323)	0.988	0.327 (0.068–1.576)	0.164	0.094 (0.005–1.720)	0.111	0.399 (0.018–6.572)	0.475	0.322	–
**Invasiveness score**	0.901 (0.440–1.846)	0.776	0.721 (0.341–1.525)	0.393	0.474 (0.201–1.119)	0.088	**0.274 (0.093–0.811)**	**0.019**	**0.002**	–
0	1.394 (0.601–3.235)	0.439	1.088 (0.426–2.780)	0.860	1.465 (0.514–4.175)	0.475	**3.848 (1.055–14.033)**	**0.041**	0.217	+
1	0.626 (0.262–1.493)	0.291	1.031 (0.348–3.051)	0.956	1.347 (0.442–4.108)	0.600	1.265 (0.307–5.206)	0.745	0.108	+
2	1.140 (0.357–3.633)	0.825	1.867 (0.693–5.026)	0.217	1.818 (0.600–5.504)	0.290	0.604 (0.151–2.423)	0.477	0.645	–
3	0.887 (0.309–2.548)	0.824	0.822 (0.303–2.229)	0.700	0.923 (0.225–3.788)	0.911	0.871 (0.171–4.443)	0.868	0.837	+
4	1.955 (0.190–20.123)	0.573	0.285 (0.033–2.457)	0.254	0.112 (0.006–2.013)	0.137	0.300 (0.016–5.717)	0.423	0.234	–
5	NA^b^	NA	0.485 (0.026–9.214)	0.630	0.339 (0.018–6.313)	0.468	0.537 (0.027–10.654)	0.683	0.837	+
**Extent of thyroid operation**										
Total thyroidectomy	3.033 (0.667–13.791)	0.151	11.624 (0.666–202.893)	0.093	0.630 (0.111–3.562)	0.601	1.741 (0.091–33.147)	0.712	0.617	–
Other										
**Lymph node dissection performed**	1.457 (0.650–3.269)	0.361	0.892 (0.384–2.069)	0.789	0.674 (0.249–1.821)	0.436	0.424 (0.126–1.424)	0.165	**0.028**	–
Level ≥6	0.896 (0.342–2.353)	0.824	**3.846 (1.208–12.195)**	**0.023**	3.663 (0.953–14.085)	0.059	3.817 (0.789–18.519)	0.096	0.227	
Level 1–5	1.116 (0.425–2.926)	0.824	**0.260 (0.082–0.828)**	**0.023**	0.273 (0.071–1.049)	0.059	0.262 (0.054–1.267)	0.096	0.227	–
**RAI performed**	1.966 (0.720–5.363)	0.187	2.058 (0.692–6.120)	0.194	0.537 (0.187–1.543)	0.249	0.898 (0.173–4.659)	0.898	0.200	–
**Follow–up**, yrs	−1.316 (−2.770–0.138)	0.076	**−3.607 (−6.485 - −0.729)**	**0.015**	**−7.295 (−10.606 - −3.985)**	**<0.001**	**−5.129 (−9.320 - −0.939)**	**0.017**	0.225	–
**LN recurrences (reoperated after 6 mo)**	3.460 (0.375–31.926)	0.274	0.305 (0.016–5.674)	0.426	1.039 (0.101–10.662)	0.974	1.743 (0.089–34.134)	0.714	0.578	–
**Recurrence–free survival**	4.571 (0.463–45.152)^c^	0.193	1.592 (0.011–29.613)^c^	0.826	14.238 (0.695–291.827)^c^	0.085	2.411 (0.017–31.533)^c^	0.654	0.863	+
**RIT response**	0.483 (0.127–1.841)	0.287	1.521 (0.339–6.820)	0.584	0.395 (0.059–2.636)	0.337	1.204 (0.135–10.755)	0.868	0.757	+
RAI–R recurrence vs. other	2.765 (0.295–25.936)	0.373	NA	NA	11.762 (0.608–227.448)	0.103	2.610 (0.119–57.105)	0.542	0.888	+
Excellent vs. other	0.564 (0.149–2.140)	0.400	1.481 (0.330–6.651)	0.608	0.402 (0.060–2.715)	0.350	1.229 (0.136–11.132)	0.854	0.772	+

a *Non-adjusted unless otherwise specified*.

b *Not available due to zero counts*.

c *The proportional hazard regression*.

*Numbers in bold indicate statistical significance*.

Radiation thyroid doses from ^131^I did not differ significantly between the BRAF^V600E^-positive and BRAF^V600E^-negative PTCs in any POC quartile, and nor did median POC estimates. No statistically significant trends for radiation doses and POC were observed, which was an expected result given the subdivision of all tumors by POC quartiles in this analysis and the direct POC proportionality to the dose.

#### Histopathological Characteristics

Comparative characteristics of PTCs with different BRAF statuses in the individual POC quartiles were generally concordant with those in the whole group, although statistical significance was not necessarily achieved in all quartiles (Table 3). The differences included the smaller tumor size (and more frequent microcarcinomas), more frequent oncocytic changes and multifocality, higher Ki67 LI, more frequent dominant papillary and less frequent follicular growth patterns, less frequent lymphatic/vascular invasion, extrathyroidal extension, regional and distant metastasis in the BRAF^V600E^-positive PTCs. Except for a downtrend for regional metastasis (*p*_trend_ = 0.002), no other statistically significant trends were observed by POC quartiles. The uptrend for Ki67 LI (*p*_trend_ = 0.072), and the downtrend for extrathyroidal extension (*p*_trend_ = 0.061) were suggestive.

The integrative invasiveness score was lower in the BRAF^V600E^-positive PTCs, and furthermore, there was a statistically significant downtrend (*p*_trend_ = 0.002), indicating that the comparative overall invasiveness of the BRAF^V600E^-positive tumors was declining with increasing POC in comparison with the BRAF^V600E^-negative tumors. This is likely due to the fact that the invasiveness score of the BRAF^V600E^-positive PTCs was not significantly changing with increasing POC quartile (OR = 1.027, *p* = 0.863), while that of the BRAF^V600E^-negative PTCs was statistically significantly increasing (OR = 1.316, *p* = 0.007; Supplementary Table 6).

#### Clinical Characteristics

The only clinical parameter displaying a statistically significant downtrend by POC quartiles was the frequency of lymph node dissections in the BRAF^V600E^-positive group as compared with the BRAF^V600E^-negative group (*p*_trend_ = 0.028; Table 3). Note that despite the absence of POC-quartile trends and of statistically significant differences in each individual POC quartile, hazard ratios for recurrence-free survival and odds ratios for RAI-R recurrences (where available) were consistently >1 for the BRAF^V600E^-positive PTCs. The lack of statistically significant differences was likely due to the small total number of recurrences and of RAI-R recurrences, which when distributed by POC quartiles did not confer sufficient power to detect those.

## Discussion

Our analysis revealed a significant association of the BRAF^V600E^ positivity with all three time-related parameters: the older age at operation, older age at exposure, and a longer period of latency. These correlations explain previous results of our and other groups who had reported either the absence or extremely low frequency of the *BRAF*^*V*600*E*^ mutation in radiogenic childhood PTC (10, 33, 42–44). Because of a longer “lag” in tumor development, patients with the BRAF^V600E^-positive PTCs are more likely to reach adolescent and adult age when they are diagnosed with thyroid cancer. The occurrence of a longer “silent” period without clinical signs of disease is, to some extent, supported by the smaller size (and the higher frequency of microcarcinomas) of the BRAF^V600E^-positive PTCs than that of the BRAF^V600E^-negative PTCs with a shorter latency.

During the past few decades, the increase over the years in frequencies of *BRAF*^*V*600*E*^ (especially in the classic papillary variant of PTC) and of smaller-sized tumors (including microcarcinomas) accompanied by the older age of patients have been reported in sporadic PTC (45–47), although the nature of this increase remains unclear. The findings of the present work in radiogenic PTC parallel these observations. Furthermore, our previous study demonstrated that the BRAF^V600E^-positive PTCs of radiogenic and sporadic etiology display a substantial similarity in their histopathological characteristics (33). It, therefore, is possible that time-related changes in the *BRAF*^*V*600*E*^ frequency seen in sporadic PTC may also take place in radiogenic tumors.

It is noteworthy that the BRAF^V600E^-positive PTCs were associated with the smaller tumor size and the higher Ki67 LI, although there was no evidence of a direct link between these two parameters in this group. Several previous works have reported elevated KI67 LI in the BRAF^V600E^-positive PTCs in adult patients (48, 49), and a positive correlation between KI67 LI and PTC size (48, 50–52). The *BRAF*^*V*600*E*^ mutation was also associated with greater tumor size [meta-analyses (18, 20), although not in all studies (24)]. Our findings support the association between the BRAF^V600E^ status and elevated Ki67 LI but, at the same time, point to the smaller size of the BRAF^V600E^-positive radiogenic tumors. These associations remained significant even after the most stringent adjustment for age factor, attesting to the credibility of the findings. A parsimonious explanation would be that a substantial proportion of the BRAF^V600E^-positive, initially rather silent, PTCs have reached the stage of more active growth when they were detected in patients of young to middle age (from 13.5 to 49.5 years, median 37.8 years) during the first 30 years after radiation exposure but did not grow to larger size yet. The vast majority of tumors in this study were not detected due to ultrasound screenings, therefore the smaller tumor size of the BRAF^V600E^-positive PTCs could not be attributed to enhanced health surveillance of the exposed population.

The BRAF^V600E^-positive PTCs were strongly associated with the papillary dominant growth pattern, which corresponds to the classical papillary variant or mixed (conventional) PTC, and were less likely to have follicular or solid-trabecular structures (see Table 1 and Supplementary Table 1). This observation is in full agreement with literature data on both radiogenic and sporadic PTCs in patients of different ages (10, 16, 33, 42, 44, 53–57). Rare histological variants were infrequent in our study (7.0% overall) although their distribution was different between the BRAF^V600E^-positive and BRAF^V600E^-negative groups. The BRAF^V600E^-positive group had more Warthin-like and tall cell variants, while the BRAF^V600E^-negative PTCs had more tumors of diffuse sclerosing variant.

A high prevalence of the *BRAF*^*V*600*E*^ mutation (~75%) in tall cells (56–59) and Warthin-like variants (56, 60, 61) was reported in sporadic PTC, and our study corroborates this. On the other hand, we did not observe BRAF^V600E^ in the diffuse sclerosing variant of PTC while literature data indicate the mutation may be expected in about 50% of such tumors (62) varying from 0 to 61% in different studies in non-exposed patients (63–67). In our opinion, the broad variation in the *BRAF*^*V*600*E*^ prevalence in diffuse sclerosing variant may be associated with the dominant growth pattern of tumor loci. The papillary-patterned ones may display a high prevalence of the *BRAF*^*V*600*E*^ mutation. However, the solid-patterned tumor loci may be driven by other oncogenes, e.g., *RET/PTC3* fusion, which, in turn, is associated with the most aggressive behavior of the diffuse sclerosing variant PTC (62, 66). In our series, all PTCs of diffuse sclerosing variant had a solid structure that possibly explains the absence of the *BRAF*^*V*600*E*^ mutation.

The analysis of tumor invasiveness did not find evidence in support of a more aggressive tumor phenotype of the BRAF^V600E^-positive radiogenic PTCs. This is in contrast to the results of a number of works on sporadic PTC, mostly in adult patients as reported in meta-analyses (18–25). We again explain this by the particular characteristics of patients in our study whose median age was relatively young. We further verified that the lower invasiveness of the BRAF^V600E^-positive tumors was not due to rare PTC variants, and obtained very similar data for common histological variants only. Tumors of rare PTC variants were more aggressive among the BRAF^V600E^-negative PTCs because of the higher frequency of the diffuse-sclerosing variant which was previously associated with childhood age and more aggressive behavior in both radiogenic and sporadic PTCs (54, 62, 68). However, the major difference in tumor invasiveness between the BRAF^V600E^-positive and the BRAF^V600E^-negative radiogenic PTCs was not determined by these tumors.

Treatment options were rather similar in the groups. Most patients (90–96%) underwent total thyroidectomy and lymph node dissection in about 50% of cases (see Table 1). The more frequent lymph node dissection in levels 1–5 in the BRAF^V600E^-negative group is likely due to the more pronounced signs of regional metastases in patients of younger age (54). Postoperative RAI treatment was performed in more than 80% of cases with excellent response achieved in 83–90% of patients.

The most clinically important observation in our study was the relationship of BRAF^V600E^ to the risk of recurrence, and of its RAI-R type. Despite the low recurrence rate (3–4%) in both groups, the BRAF^V600E^ positivity appeared to be a risk factor. Interestingly, in our earlier study of patients with Chernobyl PTC aged under 29 (median age 24), BRAF^V600E^ did not affect the chance of recurrence (33). However, in the present study in patients aged up to 50 years (median age 38), BRAF^V600E^ had a nearly independent statistically significant effect. Furthermore, all recurrences in the BRAF^V600E^-positive group were RAI-R and occurred in patients 33–46 years old with PTCs developed after the 28–30 latency period. A recent meta-analysis demonstrated that the *BRAF*^*V*600*E*^ mutation significantly increased the risk of RAI-R differentiated thyroid cancer, and also identified the *TERT* promoter mutation as an important factor for RAI-R (69, 70) which, in turn, is well-established to correlate with older patient age (71–78). It, therefore, is plausible to suggest that in radiogenic thyroid cancer, the mechanisms underlying RAI-R may also involve cooperative effects of mutations that take place in middle-aged but not in younger patients.

For practical purposes, we created several multivariate models of RAI-R recurrence onset over time after operation (see Table 2). These models indicate that larger tumor size, multifocal growth, oncocytic changes, and BRAF^V600E^-positivity may point to the elevated probability of the loss of sensitivity to RAI therapy if the disease recurs during the follow-up. Our estimates suggest that each additional millimeter of tumor size may increase the risk of developing an RAI-R recurrence by 1–10%, and multifocality, oncocytic changes, and the presence of the *BRAF*^*V*600*E*^ mutation elevate such risk severalfold at any time point after surgery as compared to the corresponding risk for primary PTCs lacking these qualitative characteristics. Tumor size, multifocality, and oncocytic changes are easily assessable on a routine pathological examination of postsurgical tissues, and the *BRAF* status can be determined using IHC or molecular methods in either preoperative biopsy or surgical material. We believe these findings might be useful for better management of radiogenic PTC.

Since demographic information and individual radiation doses were available for the study, it was possible to estimate the chance of tumor development due to radiation exposure in terms of probability of causation (POC). POC is directly proportional to the radiation dose and is also dependent on age at exposure (the younger age increases POC), duration of the period of latency (the longer latency decreases POC), and gender (modest changes for thyroid cancer). Given these effects, the BRAF^V600E^-positive PTCs displayed lower POC because of lower dose to the thyroid, older age at exposure, and longer latency period (see Table 1). Nevertheless, about 10% of the BRAF^V600E^-positive PTCs had a high POC exceeding 75%.

In view of a broad distribution of PTCs by POC, we attempted to determine whether the BRAF^V600E^ associations may display monotonic changes across POC levels. For this purpose, the BRAF^V600E^ effect sizes were calculated for different POC quartiles and assessed for the linear trends.

Many associations seen in the whole-group analysis were reproduced in the POC quartiles. For example, BRAF^V600E^ was associated with older age at operation and older age at exposure, longer latency, smaller tumor size, more frequent microcarcinomas and oncocytic changes, higher Ki67 LI, dominant papillary growth pattern, and lower invasiveness in most, although not necessarily in all POC quartiles (see Table 3). However, only a few correlations displayed statistically significant trends.

Among those, patient age at the time of exposure had a downtrend indicating that the influence of the BRAF status on this parameter was declining with increasing POC. This may also be interpreted as the diminishing difference in age at exposure between patients with the BRAF^V600E^-positive and BRAF^V600E^-negative PTCs with increasing POC.

Establishing whether tumor aggressiveness or prognosis were changing with POC level was of particular interest. The comparative frequency of lymph node involvement (N1) displayed the downtrend for association with BRAF^V600E^ with increasing POC; this held true for both central (N1a) and lateral (N1b) node metastases. Increasing POC did not significantly affect the frequencies of nodal disease in separate BRAF^V600E^-positive and BRAF^V600E^-negative groups (see Supplementary Table 6), and odds ratios were consistently <1 for the BRAF^V600E^-positive and >1 for the BRAF^V600E^-negative PTCs. These findings indicate no evidence of elevation of lymph node involvement frequency for the BRAF^V600E^-positive tumors with increasing POC.

The comparative frequency of extrathyroidal extension displayed a suggestive downtrend. We explain this by the fact that the increasing POC level was significantly associated with an increasing frequency of extrathyroidal extension in the BRAF^V600E^-negative PTCs, while no significant changes were seen for the BRAF^V600E^-positive tumors (see Supplementary Table 6). Similarly, there was a significant positive association between the invasiveness score and POC level in the BRAF^V600E^-negative PTCs, and no changes in the BRAF^V600E^-positive tumors; this resulted in a significant downtrend for the comparative invasiveness score of the BRAF^V600E^-positive PTCs. In fact, except for the increasing frequency of encapsulated tumors with increasing POC, no POC level effects were seen in the BRAF^V600E^-positive tumors for all parameters, including histopathological, clinical, and prognostic aspects (see Supplementary Table 6).

Although the major goal of this work, i.e., the assessment of BRAF^V600E^ associations with different clinicopathological characteristics and radiation exposure in radiogenic PTC, was mostly achieved, our study had some limitations. First, genetic alterations other than *BRAF*^*V*600*E*^ were not analyzed. We believe that information on those might improve our understanding of pathogenetic mechanisms of radiogenic PTC and provide additional clues about the molecular background of RAI-R tumors in middle-aged patients exposed to internal radiation. Second, although all patients in this study were aged from 0 to full 18 years at exposure, those with BRAF^V600E^-positive tumors were older at the time of the Chernobyl accident and were diagnosed later in time after the longer latency. Therefore, some birth cohort and period effects could not be ruled out. Finally, in view of the large number of statistical tests in this work, some of them may need to be considered with caution.

In conclusion, our study demonstrates that the *BRAF*^*V*600*E*^ mutation increases in frequency over time after exposure to radiation in the group of patients whose oldest age is approaching 50. Patients diagnosed during the 30 years after the Chernobyl accident with the BRAF^V600E^-positive PTC were of an older age at exposure and at surgery, were diagnosed after a longer period of latency, had lower radiation doses to the thyroid and lower POC. The BRAF^V600E^-positive PTCs were smaller in size and were strongly associated with more frequent oncocytic changes, multifocality, higher Ki67 LI and dominant papillary growth pattern. There was no evidence that BRAF^V600E^ positivity conferred a more aggressive tumor phenotype, and clinicopathological characteristics of the BRAF^V600E^-positive PTCs did not change with POC level. Thus, no particular recommendations could be issued for primary management of the BRAF^V600E^-positive PTCs in patients of middle age exposed to internal radiation. However, BRAF^V600E^ had a prognostic impact on disease-free survival and, of importance, likely increased the chance of RAIR-R recurrence. In this regard, determination of the BRAF status and availability of specific pathological features associated with BRAF^V600E^ may be beneficial for exposed patients considered for RAI therapy, and during follow-up. Further studies are necessary to establish whether BRAF^V600E^ will lead to the acquisition of more advanced clinical manifestations and further worsening of prognosis in patients exposed to Chernobyl radiation and diagnosed for PTC after even longer latency and at an older age, as documented in patients with sporadic PTC.

## Data Availability Statement

The original contributions presented in the study are included in the article/Supplementary Material, further inquiries can be directed to the corresponding author.

## Ethics Statement

The studies involving human participants were reviewed and approved by Bioethics Committee, State Institution V.P. Komisarenko Institute of Endocrinology and Metabolism of the National Academy of Medical Sciences of Ukraine, the Chernobyl Tissue Bank, Ethics Committee of Nagasaki University. Written informed consent to participate in this study was provided by the participants' legal guardian/next of kin.

## Author Contributions

LZ, TB, TIR, MI, MT, SY, NM, and VAS: study design and methodology. LZ, TB, SC, and MB: clinical and pathological data. LZ, TB, TIR, and MI: investigation and formal analysis. SM: thyroid dosimetry. TB and VAS: statistical analysis, data interpretation, and writing of the manuscript. LZ, TB, TIR, MI, MT, SY, NM, MB, SC, SM, and VAS: revision of the manuscript. All authors have reviewed the manuscript and approved the final version.

## Funding

This research was supported in part by the Program of the Network-Type Joint Usage/Research Center for Radiation Disaster Medical Science, intramurally by the Atomic Bomb Disease Institute, Nagasaki University, and the Japan Society for the Promotion of Science (JSPS), KAKENHI Grant Numbers 19K07471, 19KK02670001, and 20KK0217.

## Conflict of Interest

The authors declare that the research was conducted in the absence of any commercial or financial relationships that could be construed as a potential conflict of interest.

## Publisher's Note

All claims expressed in this article are solely those of the authors and do not necessarily represent those of their affiliated organizations, or those of the publisher, the editors and the reviewers. Any product that may be evaluated in this article, or claim that may be made by its manufacturer, is not guaranteed or endorsed by the publisher.

## Supplementary Material

Supplementary Figure 1 Violation of the proportional hazard assumption in the Cox model of the development of PTCs with different BRAF status in time after exposure (Latency, years). **(A)** The observed standardized score process and first 20 simulated realizations from the null distribution for the BRAF status. **(B)** The Schoenfeld residuals for the BRAF status variable plotted against the duration of the period of latency, and a smoothing spline. **(C)** Overlaid Kaplan-Meier and the proportional hazard model survival estimates for the BRAF status. **(D)** Overlaid Kaplan-Meier and the extended proportional hazard model with the time-dependent BRAF status.

Supplementary Figure 2 Relationship between radiation dose to the thyroid and the probability of causation in different age at exposure groups.

Supplementary Table 1 Comparative characteristics of the BRAF^V600E^-positive vs. BRAF^V600E^-negative histological PTC variants.

Supplementary Table 2 Characteristics of the primary tumors and recurrent metastases according to the BRAF status.

Supplementary Table 3 Correlation of patient age at operation with baseline, histopathological, and clinical characteristics.

Supplementary Table 4 Multivariate comparison of the BRAF^V600E^-positive vs. BRAF^V600E^-negative PTCs adjusted for sex and age at operation.

Supplementary Table 5 Comparative characteristics of the BRAF^V600E^-positive vs. BRAF^V600E^-negative PTCs in the groups matched 1:1 by age at operation (±2 years).

Supplementary Table 6 POC effects (by quartiles) on characteristics of the BRAF^V600E^-positive and BRAF^V600E^-negative PTCs.
