# Supplementary material for: Clinicopathological Implications of the BRAFV600E Mutation in Papillary Thyroid Carcinoma of Ukrainian Patients Exposed to the Chernobyl Radiation in Childhood: A Study for 30 Years After the Accident
Source: Front Med (Lausanne). 2022 Apr 26;9:882727. doi: 10.3389/fmed.2022.882727 (PMC9159157; doi:10.3389/fmed.2022.882727)
Supplement: Supplementary Figure 1 — Violation of the proportional hazard assumption in the Cox model of the development of PTCs with different BRAF status in time after exposure (Latency, years). (A) The observed standardized score process and first 20 simulated realizations from the null distribution for the BRAF status. (B) The Schoenfeld residuals for the BRAF status variable plotted against the duration of the period of latency, and a smoothing spline. (C) Overlaid Kaplan-Meier and the proportional hazard model survival estimates for the BRAF status. (D) Overlaid Kaplan-Meier and the extended proportional hazard model with the time-dependent BRAF status. [file Data_Sheet_1.docx]

Supplementary Material

# Supplementary Figures and Tables

Supplementary Figures include Supplementary Figure 1 and Supplementary Figure 2 related to subsection 3.1 Baseline and Radiation Exposure Characteristics of the main text of the article.

Supplementary Tables include Supplementary Table 1 related to subsection 3.3 Clinical Characteristics; Supplementary Table 2, Supplementary Table 3 and Supplementary Table 4 related to subsection 3.4 Clinical Characteristics; and Supplementary Table 5 related to subsection 3.5.2 Relationship of the BRAF^V600E^ Status with the POC Level/Histopathological Characteristics of the main text.

## Supplementary Figures


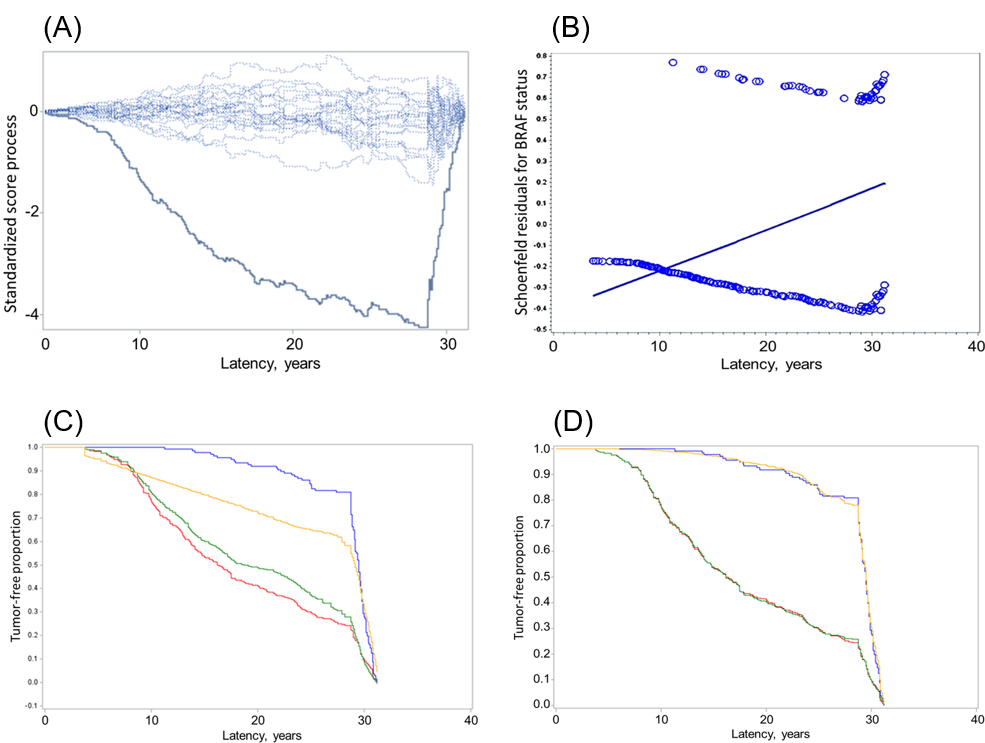


**Supplementary Figure 1**. Violation of the proportional hazard assumption in the Cox model of the development of PTCs with different BRAF status in time after exposure (Latency, years). **(A)** The observed standardized score process (solid line) and first 20 simulated realizations from the null distribution (dotted lines) for the BRAF status; a total of 1000 simulated realizations were tested (p < 0.001 by the Kolmogorov-type supremum test); the deviation of the observed process from the pattern seen for simulated realizations is a strong indicator of nonproportional hazards for the BRAF status. **(B)** The Schoenfeld residuals for the BRAF status variable plotted against the duration of the period of latency (circles) and a smoothing spline (smooth = 0.5, solid line); the non-horizontal shape of the smoothed curve is a strong indicator of nonproportional hazards for the BRAF status. **(C)** Overlaid Kaplan-Meier (blue - the BRAF^V600E^-positive, red – the BRAF^V600E^-negative PTCs) and the proportional hazard model (orange - the BRAF^V600E^-positive, green - the BRAF^V600E^-negative) survival estimates for the BRAF status; the proportional hazard model performance was poor for both the BRAF^V600E^-positive and BRAF^V600E^-negative PTCs indicative of a need for model modification. **(D)** Overlaid Kaplan-Meier (blue - the BRAF^V600E^-positive, red – the BRAF^V600E^-negative PTCs) and the extended proportional hazard model with the time-dependent BRAF status (orange - the BRAF^V600E^-positive, green - the BRAF^V600E^-negative) survival estimates for the BRAF status; the extended proportional hazard model performed well for both the BRAF^V600E^-positive and BRAF^V600E^-negative PTCs.


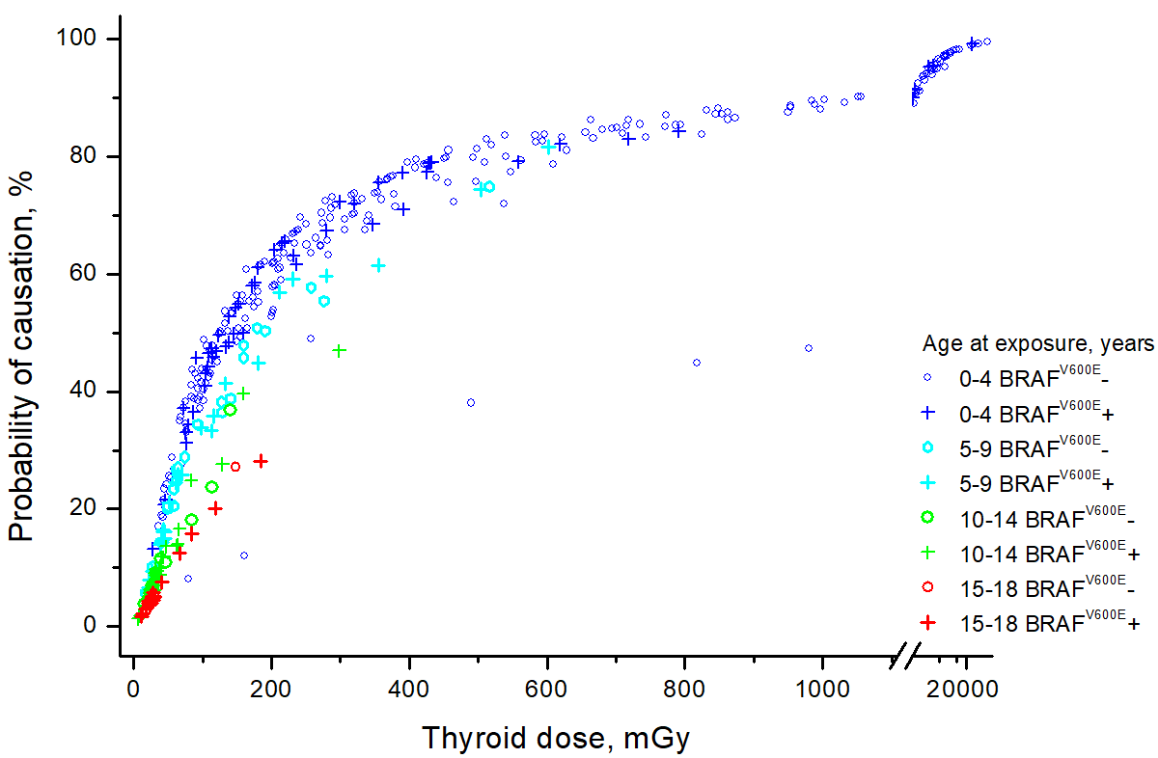


**Supplementary Figure 2.** Relationship between radiation dose to the thyroid and the probability of causation in different age at exposure groups. The BRAF^V600E^-positive PTCs are indicated by the crosses and BRAF^V600E^-negative PTCs by the circles.

## Supplementary Tables

**Supplementary Table 1.** Comparative characteristics of the BRAF^V600E^-positive versus BRAF^V600E^-negative histological PTC variants

| **Histological subtype (variant)** | **BRAF^V600E^(+) (n=136)** |  | **BRAF^V600E^(-) (n=292)** |  | **p-value** univariate |  | **OR (95%CI)**^a^ | **p-value** multivariate |
| --- | --- | --- | --- | --- | --- | --- | --- | --- |
| Classic papillary | 37 (27.2%) |  | 39 (13.4%) |  | **< 0.001** |  | **2.391 (1.437-3.977)** | **< 0.001** |
| Follicular | 11 (8.1%) |  | 72 (24.7%) |  | **< 0.001** |  | **0.282 (0.144-0.554)** | **< 0.001** |
| Solid/trabecular | 28 (20.6%) |  | 102 (34.9%) |  | **0.003** |  | **0.473 (0.292-0.766)** | **0.002** |
| Conventional PTC^b^ | 43 (31.6%) |  | 66 (22.6%) |  | 0.056 |  | **1.590 (1.008-2.510)** | **0.046** |
| Diffuse sclerosing | 0 |  | 11 (3.8%) |  | **0.020** |  | 0.076 (0.005-1.281) | 0.074 |
| Tall cell | 9 (6.6%) |  | 1 (0.3%) |  | **< 0.001** |  | **19.157 (2.351-156.100)** | **0.006** |
| Warthin-like | 8 (5.9%) |  | 0 |  | **< 0.001** |  | **36.243 (2.282-575.645)** | **0.011** |
| Hobnail | 0 |  | 1 (0.3%) |  | 1.000 |  | 0.645 (0.034-12.190) | 0.770 |

^a^ adjusted for sex

^b^ Tumors with mixed architecture essentially featuring papillary area(s)

**Supplementary Table 2**. Characteristics of the primary tumors and recurrent metastases according to the BRAF status

| **Parameters** | **BRAF^V600E^(+) (n=6)** |  | **BRAF^V600E^(-) (n=9)** |  | **p-value** univariate | | |
| --- | --- | --- | --- | --- | --- | --- | --- |
|  | number or median (% or IQR) |  | number or median (% or IQR) |  |  |  |  |
| ***Primary tumor*** | | | | | |  |  |
| **Sex** F/M (%M; F:M ratio) | 4/2 (33.3%; 2.0:1) |  | 7/2 (22.2%; 3.5:1) |  | 1.000 | |  |
| **Age at operation**, years | 39.5 (34.0-43.5) |  | 16.6 (12.1-22.2) |  | **0.003** | |  |
| **Age at exposure**, years | 9.6 (4.0-13.2) |  | 3.0 (3.0-4.0) |  | **0.008** | |  |
| **Period of latency**, years | 29.2 (29.1-30.2) |  | 15.7 (12.0-17.6) |  | **0.001** | |  |
| **Radiation dose to the thyroid**, mGy | 36 (28-44) |  | 288 (175-825) |  | **0.018** | |  |
| **Probability of Causation (POC)**, % | 11.8 (4.8-20.7) |  | 54.3 (47.4-73.2) |  | **0.026** | |  |
| ≤ 25% | 5 (83.3%) |  | 1 (11.1%) |  | **0.011** | |  |
| > 25 – 50% | 0 |  | 3 (33.3%) |  | 0.229 | |  |
| > 50 – 75% | 1 (16.7%) |  | 3 (33.3%) |  | 0.604 | |  |
| > 75 – 100% | 0 |  | 2 (22.2%) |  | 0.486 | |  |
| **Tumor size**, mm | 27 (24-33) |  | 23 (16-37) |  | 1.000 | |  |
| ≤10 mm (microcarcinoma) | 0 |  | 1 (11.1%) |  | 1.000 | |  |
| 11 – 20 mm | 1 (16.7%) |  | 3 (33.3%) |  | 0.604 | |  |
| 21 – 40 mm | 5 (83.3%) |  | 3 (33.3%) |  | 0.119 | |  |
| 40+ mm | 0 |  | 2 (22.2%) |  | 0.486 | |  |
| **Complete tumor capsule** | 0 |  | 0 |  | NA^a^ | |  |
| **Dominant growth pattern** |  |  |  |  | 1.000 | |  |
| papillary | 4 (66.7%) |  | 6 (66.7%) |  | 1.000 | |  |
| follicular | 0 |  | 0 |  | NA | |  |
| solid-trabecular | 2 (33.3%) |  | 3 (33.3%) |  | 1.000 | |  |
| **Ki67 labeling index** | 4.9 (3.8-5.6) |  | n=8; 4.5 (2.5-9.4) |  | 0.852 | |  |
| Ki67 group | 1.5 (1-2) |  | 1 (1-2.5) |  | 1.000^b^ | |  |
| 0 – 5% | 3 (50.0%) |  | 5 (62.5%) |  | 1.000 | |  |
| > 5 – 10% | 3 (50.0%) |  | 1 (12.5%) |  | 0.245 | |  |
| > 10% | 0 |  | 2 (25.0%) |  | 0.473 | |  |
| **Oncocytic changes** | 6 (100%) |  | 4 (44.4%) |  | **0.044** | |  |
| **Multifocality** | 5 (83.3%) |  | 2 (22.2%) |  | **0.041** | |  |
| **Lymphatic/vascular invasion** | 3 (50%) |  | 8 (88.9%) |  | 0.235 | |  |
| **Extrathyroidal extension (any)** | 4 (66.7%) |  | 9 (100%) |  | 0.143 | |  |
| **N category (N1)** | 5 (83.3%) |  | 6 (66.7%) |  | 0.604 | |  |
| N1a | 2 (33.3%) |  | 3 (33.3%) |  | 1.000 | |  |
| N1b | 3 (50.0%) |  | 3 (33.3%) |  | 0.622 | |  |
| **M category (M1)** | 0 |  | 3 (33.3%) |  | 0.229 | |  |
| **pT category (8 Ed)** |  |  |  |  | 0.514^b^ | |  |
| pT1 | 1 (16.7%) |  | 3 (33.3%) |  | 0.604 | |  |
| pT1a | 0.0 |  | 1 (11.1%) |  | 1.000 | |  |
| pT1b | 1 (16.7%) |  | 2 (22.2%) |  | 1.000 | |  |
| pT2 | 5 (83.3%) |  | 1 (11.1%) |  | **0.011** | |  |
| pT3 | 0 |  | 5 (55.6%) |  | **0.044** | |  |
| pT3a | 0 |  | 2 (22.2%) |  | 0.486 | |  |
| pT3b | 0 |  | 3 (33.3%) |  | 0.229 | |  |
| **Invasiveness score (any Ex)** | 3 (2-4) |  | 3 (2-4) |  | 0.795^b^ | |  |
| 0 | 0 |  | 0 |  | NA | |  |
| 1 | 1 (16.7%) |  | 0 |  | 0.400 | |  |
| 2 | 1 (16.7%) |  | 3 (33.3%) |  | 0.604 | |  |
| 3 | 2 (33.3%) |  | 2 (22.2%) |  | 1.000 | |  |
| 4 | 2 (33.3%) |  | 4 (44.4%) |  | 1.000 | |  |
| 5 | 0 |  | 0 |  | NA | |  |
| **Thyroid surgery volume** |  |  |  |  | 1.000 | |  |
| total thyroidectomy | 6 (100%) |  | 8 (88.9%) |  |  | |  |
| other | 0 |  | 1 (11.1%) |  |  | |  |
| **Lymph node dissection performed** | 5 (83.3%) |  | 7 (77.8%) |  | 1.000 | |  |
| level ≥ 6 | 2 (33.3%) |  | 5 (55.6%) |  | 0.329 | |  |
| level 1 – 5 | 3 (50.0%) |  | 2 (22.2%) |  | 0.329 | |  |
| **RIT performed** | 6 (100%) |  | 8 (88.9%) |  | 1.000 | |  |
| **RIT cycles** | 2 (1-2) |  | 3 (1-3) |  | 0.205 | |  |
| **Cumulative RI activity,** MBq | 7830 (4360-10752) |  | 8282 (2779-16894) |  | 0.776 | |  |
| ***Recurrent metastasis*** | | | | | |  |  |
| **Time between 1st surgery and recurrence**, yrs | 1.7 (0.9-2.8) |  | 2.2 (1.0-5.4) |  | 0.388 | |  |
| **Dominant growth pattern** |  |  |  |  | 0.633 | |  |
| papillary | 4 (66.7%) |  | 5 (55.6%) |  | 0.567 | |  |
| follicular | 0 |  | 0 |  | NA | |  |
| solid-trabecular | 2 (33.3%) |  | 4 (44.4%) |  | 0.567 | |  |
| **Ki67 labeling index** | n=5; 3.3 (3.0-4.0) |  | n=4; 5.8 (3.7-7.9) |  | 0.413 | |  |
| **Oncocytic changes** | 6 (100.0%) |  | 4 (44.4%) |  | **0.044** | |  |
| **Cystic changes** | 2 (33.3%) |  | 1 (11.1%) |  | 0.525 | |  |
| **RIT recurrence response** | n=6 |  | n=8 |  |  | |  |
| RAI-R recurrence *vs* other | 6 (100%) |  | 2 (25.0%) |  | **0.010** | |  |
| excellent *vs* other | 0 |  | 5 (62.5%) |  | **0.031** | |  |

^a^ Not available

^b^ The trend test (the Cochran-Armitage test)

**Supplementary Table 3**. Correlation of patient age at operation with baseline, histopathological and clinical characteristics

| **Parameters** | **OR, b or HR (95%CI)**^a^ | **p-value** |
| --- | --- | --- |
|  | multivariate logistic, linear or proportional hazard regression | |
| **BRAF status** | **1.137 (1.109-1.167)** | **< 0.001** |
| **Sex** | **0.977 (0.960-0.994)** | **0.010** |
| **Age at exposure**, years | **0.315 (0.285-0.344)** | **< 0.001** |
| **Period of latency**, years | **1.334 (1.269-1.372)** | **< 0.001** |
| **Radiation dose to the thyroid**, mGy | **-0.171 (-0.191- -0.150)** | **< 0.001** |
| **Probability of Causation (POC),** % | **-7.135 (-7.914- -6.357)** | **< 0.001** |
| ≤ 25% | **1.259 (1.200-1.320)** | **< 0.001** |
| > 25 – 50% | 1.000 (0.982-1.018) | 0.964 |
| > 50 – 75% | **0.943 (0.924-0.963)** | **< 0.001** |
| > 75 – 100% | **0.917 (0.895-0.939)** | **< 0.001** |
| **Tumor size**, mm | **-0.105 (-0.137- -0.073)** | **< 0.001** |
| ≤10 mm (microcarcinoma) | **1.059 (1.039-1.079)** | **< 0.001** |
| 11 – 20 mm | 0.986 (0.970-1.002) | 0.081 |
| 21 – 40 mm | 0.981 (0.961-1.001) | 0.057 |
| 40+ mm | **0.929 (0.894-0.965)** | **< 0.001** |
| **Oncocytic changes** | **1.084 (1.063-1.106)** | **< 0.001** |
| **Multifocality** | 1.044 (1.023-1.065) | **< 0.001** |
| **Ki67 labeling index** | **0.137 (0.105-0.168)** | **< 0.001** |
| Ki67 group | **1.037 (1.018-1.056)** | **< 0.001** |
| 0 – 5% | **0.963 (0.946-0.981)** | **< 0.001** |
| > 5 – 10% | **1.037 (1.017-1.057)** | **< 0.001** |
| > 10% | 1.021 (0.985-1.059) | 0.259 |
| **Complete tumor capsule** | **0.960 (0.931-0.990)** | **0.009** |
| **Dominant growth pattern** | **0.965 (0.951-0.980)** | **< 0.001** |
| papillary | **1.050 (1.032-1.069)** | **< 0.001** |
| follicular | **0.978 (0.960-0.996)** | **0.015** |
| solid-trabecular | **0.975 (0.960-0.991)** | **0.003** |
| **Lymphatic/vascular invasion** | **0.933 (0.917-0.950)** | **< 0.001** |
| **Extrathyroidal extension (any)** | **0.942 (0.926-0.959)** | **< 0.001** |
| **N category (N1)** | **0.955 (0.939-0.971)** | **< 0.001** |
| N1a | 0.984 (0.963-1.005) | 0.132 |
| N1b | **0.955 (0.936-0.973)** | **< 0.001** |
| **M category (M1)** | **0.900 (0.865-0.936)** | **< 0.001** |
| **pT category (8 Ed)**  **pT category (8 Ed)** | **0.951 (0.933-0.968)** | **4.08E-08** |
| pT1 | **1.048 (1.029-1.067)** | **< 0.001** |
| pT1a | **1.058 (1.038-1.079)** | **< 0.001** |
| pT1b | 0.995 (0.979-1.011) | 0.517 |
| pT2 | 0.990 (0.969-1.011) | 0.351 |
| pT3 | **0.931 (0.906-0.956)** | **< 0.001** |
| pT3a | **0.937 (0.896-0.980)** | **0.004** |
| pT3b | **0.935 (0.907-0.965)** | **< 0.001** |
| **Invasiveness score (any Ex)** | **0.949 (0.936-0.963)** | **< 0.001** |
| 0 | **1.046 (1.027-1.066)** | **< 0.001** |
| 1 | **1.032 (1.012-1.053)** | **0.001** |
| 2 | **0.972 (0.954-0.992)** | **0.005** |
| 3 | 0.982 (0.962-1.002) | 0.077 |
| 4 | **0.947 (0.917-0.979)** | **0.001** |
| 5 | **0.865 (0.788-0.950)** | **0.002** |
| **Extent of thyroid operation** |  |  |
| total thyroidectomy | 0.998 (0.969-1.028) | 0.909 |
| other |  |  |
| **Lymph node dissection performed** | **0.969 (0.953-0.984)** | **< 0.001** |
| level ≥ 6 | **1.053 (1.033-1.073)** | **< 0.001** |
| level 1 – 5 | **0.950 (0.932-0.968)** | **< 0.001** |
| **RAI treatment performed** | 0.988 (0.967-1.009) | 0.256 |
| **Follow-up**, yrs | **-1.961 (-2.124- -1.798)** | **< 0.001** |
| **LN recurrences (reoperated after 6 mo)** | 1.003 (0.962-1.047) | 0.873 |
| **Recurrence-free survival** | **1.103 (1.054-1.155)** | **< 0.001** |
| **RAI treatment response** | 0.973 (0.430-2.203) | 0.948 |
| RAI-R tumor or recurrence *vs* other | **1.094 (1.0132-1.180)** | **0.021** |
| excellent *vs* other | **0.953 (0.925-0.982)** | **0.002** |

**Supplementary Table 4**. Multivariate comparison of the BRAF^V600E^-positive (n=136) *versus* BRAF^V600E^-negative (n=292) PTCs adjusted for sex and age at operation

| **Parameters** | **OR, b or HR (95%CI)**^a^ | **p-value** |
| --- | --- | --- |
|  | multivariate logistic, linear or proportional hazard regression | |
| **Sex** | 0.794 (0.455-1.387)^b^ | 0.418 |
| **Age at operation**, years | **14.346 (12.293-16.400)**^c^ | **< 0.001** |
| **Age at exposure**, years | **4.858 (3.959-5.757)**^c^ | **< 0.001** |
| **Period of latency**, years | **9.681 (8.165-11.197)**^c^ | **< 0.001** |
| Period of latency^d^ | **0.085 (0.023-0.315)** | **< 0.001** |
| BRAF status*latency^d^ | **1.100 (1.048-1.154)** | **< 0.001** |
| **Radiation dose to the thyroid**, mGy | -144.573 (-619.431 - 330.285) | 0.550 |
| **Probability of Causation (POC),** % | **-27.534 (-33.126- -21.941)**^e^ | **< 0.001** |
| ≤ 25% | **6.884 (4.265-11.111)**^e^ | **< 0.001** |
| > 25 – 50% | 0.890 (0.553-1.432)^e^ | 0.632 |
| > 50 – 75% | **0.470 (0.279-0.792)**^e^ | **0.005** |
| > 75 – 100% | **0.223 (0.120-0.415)**^e^ | **< 0.001** |
| **Tumor size**, mm | **-4.322 (-7.325- -1.318)** | **0.005** |
| ≤10 mm (microcarcinoma) | **3.148 (1.842-5.382)** | **< 0.001** |
| 11 – 20 mm | **0.557 (0.337-0.923)** | **0.023** |
| 21 – 40 mm | 0.620 (0.322-1.193) | 0.152 |
| 40+ mm | 0.291 (0.060-1.406) | 0.125 |
| **Oncocytic changes** | **2.233 (1.331-3.747)** | **0.002** |
| **Multifocality** | 1.683 (0.944-3.002) | 0.078 |
| **Ki67 labeling index** | **1.168 (0.454-1.881)** | **0.001** |
| Ki67 group | **1.799 (1.067-3.034)** | **0.028** |
| 0 – 5% | **0.537 (0.317-0.911)** | **0.021** |
| > 5 – 10% | **1.879 (1.075-3.285)** | **0.027** |
| > 10% | 1.248 (0.429-3.634) | 0.685 |
| **Complete tumor capsule** | 0.456 (0.152-1.373) | 0.162 |
| **Dominant growth pattern** | **0.410 (0.258-0.650)** | **< 0.001** |
| papillary | **3.555 (2.103-6.011)** | **< 0.001** |
| follicular | **0.285 (0.149-0.545)** | **< 0.001** |
| solid-trabecular | 0.686 (0.410-1.149) | 0.152 |
| **Lymphatic/vascular invasion** | 0.654 (0.393-1.090) | 0.103 |
| **Extrathyroidal extension (any)** | 0.912 (0.531-1.567) | 0.739 |
| **N category (N1)** | 0.966 (0.575-1.622) | 0.895 |
| N1a | 1.352 (0.690-2.674) | 0.380 |
| N1b | 0.733 (0.394-1.362) | 0.325 |
| **M category (M1)** | 0.323 (0.069-1.533) | 0.155 |
| **pT category (8 Ed)** | **0.520 (0.288-0.938)** | **0.030** |
| pT1 | **1.919 (1.062-3.470)** | **0.031** |
| pT1a | **3.174 (1.856-5.429)** | **< 0.001** |
| pT1b | **0.566 (0.339-0.942)** | **0.029** |
| pT2 | 0.689 (0.342-1.385) | 0.295 |
| pT3 | 0.389 (0.147-1.028) | 0.057 |
| pT3a | 0.460 (0.088-2.418) | 0.359 |
| pT3b | 0.378 (0.118-1.219) | 0.103 |
| **Invasiveness score (any Ex)** | 0.960 (0.618-1.493) | 0.857 |
| 0 | 1.108 (0.647-1.898) | 0.708 |
| 1 | 0.690 (0.385-1.236) | 0.212 |
| 2 | 1.557 (0.833-2.912) | 0.165 |
| 3 | 1.160 (0.599-2.245) | 0.660 |
| 4 | 0.443 (0.133-1.473) | 0.184 |
| 5 | 0.425 (0.023-7.996) | 0.568 |
| **Follow-up**, yrs | 0.421 (-0.776-1.619) | 0.490 |
| **Extent of thyroid operation** |  |  |
| total thyroidectomy | **3.951 (1.348-11.582)** | **0.012** |
| other | **0.253 (0.086-0.742)** | **0.012** |
| **Lymph node dissection performed** | 1.302 (0.788-2.150) | 0.303 |
| level ≥ 6 | 1.341 (0.740-2.428) | 0.333 |
| level 1 – 5 | 0.746 (0.412-1.350) | 0.333 |
| **RAI treatment performed** | 1.410 (0.759-2.616) | 0.277 |
| **RAI treatment response** | 0.973 (0.430-2.203) | 0.948 |
| RAI-R tumor or recurrence *vs* other | 2.937 (0.435-19.810) | 0.269 |
| excellent *vs* other | 1.042 (0.457-2.376) | 0.923 |
| **LN recurrences (reoperated after 6 mo)** | 1.593 (0.428-5.927) | 0.487 |
| **Recurrence-free survival** | 0.997 (0.166-6.401)^f^ | 0.997 |

^a^ Adjusted for sex and age at operation unless otherwise specified

^b^ Adjusted for age at operation

^c^ Adjusted for sex

^d^ The extended proportional hazard regression

^e^ Not adjusted for sex and age at operation because sex, year of birth and year of diagnosis (that determine the age at operation) are the variables included in POC calculation (see Materials and methods, Probability of causation (POC) from radiation)

^f^ The Firth’s-penalized proportional hazard model adjusted for sex, age at operation, tumor size, N and M category, extent of thyroid operation, lymph node dissection and RAI treatment

**Supplementary Table 5.** Comparative characteristics of the BRAF^V600E^-positive *versus* BRAF^V600E^-negative PTCs in the groups matched 1:1 by age at operation (±2 years)

| **Parameters** | **BRAF^V600E^(+) (n=100)** |  | **BRAF^V600E^(-) (n=100)** |  | **p-value**^a^ | **OR or b (95%CI)**^b^ | **p-value** | |
| --- | --- | --- | --- | --- | --- | --- | --- | --- |
|  | number or median (% or IQR) |  | number or median (% or IQR) |  | univariate | multivariate conditional logistic or proportional hazard, or linear regression | |  |
| **Sex** F/M (%M; F:M ratio) | 76/24 (24.0%; 3.2:1) |  | 76/24 (24.0%; 3.2:1) |  | 1.000 | 0.862 (0.382-1.944)^c^ | 0.720 | |
| **Age at operation**, years | 34.0 (28.6-38.7) |  | 33.9 (28.3-38.5) |  | **< 0.001** | 0.445 (-1.848-2.738)^d.e^ | 0.702 | |
| **Age at exposure**, years | 4.3 (2.0-9.0) |  | 4.0 (2.0-8.6) |  | **< 0.001** | 0.418 (-1.019-1.855)^d.e^ | 0.567 | |
| **Period of latency**, years | 29.4 (26.4-29.9) |  | 29.4 (26.2-30.2) |  | 0.825 | 0.121 (-1.184-1.426)^d.e^ | 0.855 | |
| **Radiation dose to the thyroid**, mGy | 114.1 (44.0-257.6) |  | 127.8 (49.5-283.0) |  | 0.142 | -80.986 (-403.497-241.524)^d^ | 0.621 | |
| **Probability of Causation (POC)**, % | 43.6 (14.8-63.6) |  | 43.9 (19.3-70.0) |  | 0.132 | -2.178 (-7.814-3.459) | 0.447 | |
| ≤ 25% | 37 (37.0%) |  | 31 (31.0%) |  | 0.456 | 0.988 (0.295-3.307) | 0.985 | |
| > 25 – 50% | 28 (28.0%) |  | 31 (31.0%) |  | 0.757 | 0.843 (0.359-1.977) | 0.873 | |
| > 50 – 75% | 22 (22.0%) |  | 21 (21.0%) |  | 1.000 | 1.010 (0.423-2.412) | 0.840 | |
| > 75 – 100% | 13 (13.0%) |  | 17 (17.0%) |  | 0.553 | 0.911 (0.327-2.536) | 0.858 | |
| **Tumor size**, mm | 11 (7-19) |  | 15 (12-24) |  | **< 0.001** | **-4.939 (-7.948- -1.930)**^d^ | **0.001** | |
| ≤10 mm (microcarcinoma) | 48 (48.0%) |  | 20 (20.0%) |  | **< 0.001** | **4.370 (1.796-10.632)** | **0.001** | |
| 11 – 20 mm | 35 (35.0%) |  | 50 (50.0%) |  | **0.044** | 0.604 (0.305-1.194) | 0.147 | |
| 21 – 40 mm | 15 (15.0%) |  | 25 (25.0%) |  | 0.121 | 0.520 (0.229-1.181) | 0.118 | |
| 40+ mm | 2 (2.0%) |  | 5 (5/0%) |  | 0.453 | 0.188 (0.015-2.328) | 0.193 | |
| **Complete tumor capsule** | 5 (5/0%) |  | 8 (8/0%) |  | 0.508 | 0.740 (0.114-4.813) | 0.753 | |
| **Dominant growth pattern** |  |  |  |  | **< 0.001** | NA^f^ | NA | |
| papillary | 59 (59.0%) |  | 30 (30.0%) |  | **< 0.001** | **3.464 (1.498-8.010)** | **0.004** | |
| follicular | 12 (12.0%) |  | 34 (34.0%) |  | **< 0.001** | 0.319 (0.125-0.813) | 0.017 | |
| solid-trabecular | 29 (29.0%) |  | 36 (36.0%) |  | 0.360 | 0.674 (0.313-1.455) | 0.315 | |
| **Ki67 labeling index** | 4.6 (3.6-6.3) |  | 3.5 (2.3-6.0) |  | 0.057 | **0.858 (0.019-1.698)**^d^ | **0.045** | |
| Ki67 group | 1 (1-2) |  | 1 (1-2) |  | 0.127 | NA | NA | |
| 0 – 5% | 57 (57.0%) |  | 68 (68.0%) |  | 0.068 | 0.734 (0.353-1.527) | 0.408 | |
| > 5 – 10% | 36 (36.0%) |  | 25 (25.0%) |  | **0.049** | 1.731 (0.788-3.902) | 0.172 | |
| > 10% | 7 (7.0%) |  | 7 (7.0%) |  | 0.605 | 0.277 (0.042-1.812) | 0.180 | |
| **Oncocytic changes** | 54 (54.0%) |  | 39 (39.0%) |  | 0.063 | 1.220 (0.645-2.305) | 0.541 | |
| **Multifocality** | 32 (32.0%) |  | 21 (21.0%) |  | 0.108 | 1.752 (0.756-4.059) | 0.191 | |
| **Lymphatic/vascular invasion** | 37 (37.0%) |  | 45 (45.0%) |  | 0.312 | 0.640 (0.317-1.292) | 0.213 | |
| **Extrathyroidal extension (any)** | 29 (29.0%) |  | 34 (34.0%) |  | 0.533 | 0.640 (0.291-1.409) | 0.268 | |
| **N category (N1)** | 36 (36.0%) |  | 37 (37.0%) |  | 1.000 | 0.914 (0.458-1.822) | 0.798 | |
| N1a | 17 (17.0%) |  | 16 (16.0%) |  | 1.000 | 0.937 (0.381-2.303) | 0.887 | |
| N1b | 19 (19.0%) |  | 21 (21.0%) |  | 0.868 | 0.932 (0.420-2.070) | 0.863 | |
| **M category (M1)** | 2 (2.0%) |  | 8 (8/0%) |  | 0.109 | 0.308 (0.043-2.191) | 0.240 | |
| **pT category (8 Ed)** |  |  |  |  | 0.147 | NA | NA | |
| pT1 | 82 (82.0%) |  | 68 (68.0%) |  | **0.038** | 2.120 (0.989-4.547) | 0.054 | |
| pT1a | 48 (48.0%) |  | 20 (20.0%) |  | **< 0.001** | **4.367 (1.780-10.713)** | **0.001** | |
| pT1b | 34 (34.0%) |  | 48 (48.0%) |  | 0.054 | 0.596 (0.298-1.192) | 0.143 | |
| pT2 | 13 (13.0%) |  | 20 (20.0%) |  | 0.265 | 0.698 (0.300-1.627) | 0.405 | |
| pT3 | 5 (5/0%) |  | 12 (12.0%) |  | 0.143 | 0.326 (0.095-1.117) | 0.075 | |
| pT3a | 2 (2.0%) |  | 2 (2.0%) |  | 1.000 | 0.421 (0.028-6.297) | 0.531 | |
| pT3b | 3 (3.0%) |  | 10 (10.0%) |  | 0.092 | 0.335 (0.084-1.335) | 0.121 | |
| **Invasiveness score (any Ex)** | 1 (0-2) |  | 1 (0-2) |  | 0.070 | NA | NA | |
| 0 | 33 (33.0%) |  | 33 (33.0%) |  | 1.000 | 1.243 (0.577-2.678) | 0.578 | |
| 1 | 22 (22.0%) |  | 25 (25.0%) |  | 0.736 | 0.748 (0.340-1.643) | 0.469 | |
| 2 | 24 (24.0%) |  | 18 (18.0%) |  | 0.392 | 1.406 (0.607-3.255) | 0.426 | |
| 3 | 18 (18.0%) |  | 15 (15.0%) |  | 0.678 | 1.182 (0.407-3.437) | 0.758 | |
| 4 | 3 (3.0%) |  | 8 (8/0%) |  | 0.227 | 0.252 (0.046-1.368) | 0.110 | |
| 5 | 0 (0.0%) |  | 1 (1.0%) |  | 1.000 | NAv^g^ | NAv | |
| **Follow-up**, yrs | 3.9 (2.8-5.4) |  | 3.4 (1.9-5.1) |  | 0.184 | 0.520 (-0.397-1.418)^d^ | 0.255 | |
| **Thyroid surgery volume** |  |  |  |  | 0.077 |  |  | |
| total thyroidectomy | 96 (96.0%) |  | 88 (88.0%) |  |  | 2.703 (0.401-18.242) | 0.307 | |
| other | 4 (4.0%) |  | 12 (12.0%) |  |  | 0.370 (0.055-2.494) | 0.307 | |
| **Lymph node dissection performed** | 53 (53.0%) |  | 46 (46.0%) |  | 0.450 | 1.099 (0.574-2.104) | 0.777 | |
| level ≥ 6 | 32 (32.0%) |  | 22 (22.0%) |  | 0.755 | 1.010 (0.476-2.146) | 0.979 | |
| level 1 – 5 | 21 (21.0%) |  | 24 (24.0%) |  | 0.755 | 0.990 (0.466-2.101) | 0.979 | |
| **RIT performed** | 80 (80.0%) |  | 76 (76.0%) |  | 0.618 | 1.068 (0.456-2.504) | 0.880 | |
| **RIT response** | n=80 |  | n=76 |  |  | 0.797 (0.526-13.209) | 0.286 | |
| RAI-R tumor or recurrence *vs* other | 6 (7.5%) |  | 2 (2.6%) |  | 0.289 | 7.748 (0.463-129.723) | 0.154 | |
| excellent *vs* other | 67 (83.8%) |  | 61 (80.3%) |  | 1.000 | 0.977 (0.335-2.852) | 0.967 | |
| **LN recurrences (reoperated after 6 mo)** | n=98; 6 (6.1%) |  | n=94; 4 (4.3%) |  | 0.377 | 1.735 (0.390-7.720) | 0.470 | |
| **Recurrence-free survival** | n=98 |  | n=94 |  | 0.866 | 0.549 (0.031-9.644)^h^ | 0.682 | |

^a^ Related samples tests (Wilcoxon signed rank test for continuous data; McNemar test for count data; Bowker test for categorical variables with several response levels; clustered log-rank test for the Period of latency and Recurrence-free survival)

^b^ Adjusted for sex and age at operation to account for residual confounding unless otherwise specified

^c^ Adjusted for age at operation

^d^ Linear regression

^e^ Adjusted for sex

^f^ Not applicable

^g^ Not available

^h^ Conditional proportional hazard regression

**Supplementary Table 6.** POC effects (by quartiles) on characteristics of the BRAF^V600E^-positive and BRAF^V600E^-negative PTCs

|  | **BRAF^V600E^(+) (n=136)** | |  | **BRAF^V600E^(-) (n=292)** | |
| --- | --- | --- | --- | --- | --- |
| **Parameters** | **OR, b or HR (95%CI)**^a^ | **p-value** |  | **OR, b or HR (95%CI)**^a^ | **p-value** |
|  | logistic, linear or proportional hazard regression | |  | logistic, linear or proportional hazard regression | |
| **Tumor size**, mm | -0.065 (-0.214-0.083) | 0.387 |  | -0.062 (-0.170- 0.045) | 0.255 |
| ≤10 mm (microcarcinoma) | 1.014 (0.728-1.413) | 0.933 |  | 1.041 (0.774-1.401) | 0.791 |
| 11 – 20 mm | 1.178 (0.835-1.661) | 0.351 |  | 1.185 (0.947-1.484) | 0.137 |
| 21 – 40 mm | 0.597 (0.326-1.092) | 0.094 |  | **0.741 (0.570-0.964)** | **0.026** |
| 40+ mm | 1.735 (0.509-5.916) | 0.379 |  | 1.068 (0.737-1.546) | 0.728 |
| **Oncocytic changes** | 0.841 (0.602-1.175) | 0.310 |  | **0.711 (0.541-0.933)** | **0.014** |
| **Multifocality** | 0.988 (0.691-1.412) | 0.948 |  | 1.186 (0.861-1.633) | 0.297 |
| **Ki67 labeling index** | 0.079 (-0.045-0.202) | 0.210 |  | -0.106 (-0.221-0.009) | 0.070 |
| Ki67 group | 1.279 (0.922-1.774) | 0.140 |  | 0.819 (0.625-1.074) | 0.149 |
| 0 – 5% | 0.780 (0.557-1.094) | 0.150 |  | 1.226 (0.934-1.610) | 0.142 |
| > 5 – 10% | 1.208 (0.857-1.702) | 0.281 |  | 0.813 (0.605-1.091) | 0.168 |
| > 10% | 1.283 (0.690-2.385) | 0.431 |  | 0.893 (0.513-1.556) | 0.690 |
| **Complete tumor capsule** | **2.730 (1.145-6.511)** | **0.023** |  | 0.944 (0.662-1.345) | 0.750 |
| **Dominant growth pattern** | 0.924 (0.667-1.279) | 0.632 |  | 1.047 (0.850-1.289) | 0.666 |
| papillary | 1.083 (0.774-1.516) | 0.641 |  | 0.823 (0.627-1.080) | 0.160 |
| follicular | 0.976 (0.588-1.617) | 0.924 |  | 1.193 (0.938-1.517) | 0.151 |
| solid-trabecular | 0.924 (0.643-1.327) | 0.668 |  | 0.976 (0.781-1.221) | 0.834 |
| **Lymphatic/vascular invasion** | 1.146 (0.813-1.615) | 0.436 |  | **1.311 (1.037-1.658)** | **0.024** |
| **Extrathyroidal extension (any)** | 1.026 (0.708-1.486) | 0.892 |  | **1.323 (1.054-1.661)** | **0.016** |
| **N category (N1)** | 0.788 (0.543-1.142) | 0.208 |  | 1.220 (0.974-1.528) | 0.083 |
| N1a | 0.830 (0.518-1.330) | 0.438 |  | 1.094 (0.813-1.471) | 0.554 |
| N1b | 0.816 (0.503-1.324) | 0.410 |  | 1.186 (0.930-1.511) | 0.169 |
| **M category (M1)** | 2.629 (0.693-9.977) | 0.156 |  | 1.364 (0.970-1.919) | 0.075 |
| **pT category (8 Ed)** | 0.749 (0.458-1.226) | 0.250 |  | 0.955 (0.765-1.192) | 0.683 |
| pT1 | 1.361 (0.828-2.238) | 0.224 |  | 1.114 (0.886-1.400) | 0.355 |
| pT1a | 1.044 (0.749-1.455) | 0.800 |  | 1.041 (0.774-1.401) | 0.791 |
| pT1b | 1.134 (0.801-1.605) | 0.479 |  | 1.084 (0.866-1.358) | 0.480 |
| pT2 | 0.661 (0.362-1.207) | 0.178 |  | **0.724 (0.542-0.968)** | **0.029** |
| pT3 | 0.988 (0.439-2.223) | 0.976 |  | 1.147 (0.868-1.516) | 0.335 |
| pT3a | 1.735 (0.509-5.916) | 0.379 |  | 1.096 (0.693-1.734) | 0.694 |
| pT3b | 0.655 (0.192-2.230) | 0.499 |  | 1.149 (0.831-1.589) | 0.401 |
| **Invasiveness score (any Ex)** | 1.027 (0.762-1.383) | 0.863 |  | **1.316 (1.078-1.606)** | **0.007** |
| 0 | 0.921 (0.648-1.309) | 0.647 |  | **0.689 (0.525-0.904)** | **0.007** |
| 1 | 0.998 (0.675-1.475) | 0.991 |  | 0.813 (0.617-1.070) | 0.140 |
| 2 | 1.250 (0.847-1.845) | 0.261 |  | **1.642 (1.226-2.200)** | **< 0.001** |
| 3 | 0.977 (0.626-1.525) | 0.919 |  | 0.857 (0.650-1.131) | 0.275 |
| 4 | 0.380 (0.066-2.195) | 0.280 |  | 1.284 (0.883-1.868) | 0.190 |
| 5 | NA^b^ | NA |  | 1.649 (0.862-3.155) | 0.131 |
| **Extent of thyroid operation** |  |  |  |  |  |
| total thyroidectomy | 1.048 (0.427-2.574) | 0.918 |  | **1.463 (1.004-2.132)** | **0.047** |
| other | 0.954 (0.389-2.342) | 0.918 |  | **0.684 (0.469-0.966)** | **0.047** |
| **Lymph node dissection performed** | 0.896 (0.642-1.251) | 0.520 |  | 1.207 (0.963-1.513) | 0.102 |
| level ≥ 6 | 1.307 (0.816-2.092) | 0.266 |  | 0.793 (0.626-1.005) | 0.055 |
| level 1 – 5 | 0.765 (0.478-1.226) | 0.266 |  | 1.261 (0.995-1.598) | 0.055 |
| **RAI performed** | 0.803 (0.530-1.216) | 0.299 |  | 1.278 (0.980-1.668) | 0.071 |
| **LN recurrences (reoperated after 6 mo)** | 0.473 (0.138-1.625) | 0.234 |  | 0.877 (0.465-1.655) | 0.686 |
| **Recurrence-free survival**^c^ | 0.376 (0.103-1.375) | 0.139 |  | 0.670 (0.312-1.437) | 0.304 |
| **RIT response** | 1.441 (0.803-2.585) | 0.220 |  | 1.156 (0.752-1.775) | 0.509 |
| RAI-R tumor or recurrence *vs* other | 0.496 (0.144-1.708) | 0.267 |  | 0.717 (0.193-2.666) | 0.619 |
| excellent *vs* other | 1.393 (0.782-2.482) | 0.261 |  | 1.165 (0.758-1.790) | 0.485 |

^a^ Non-adjusted

^b^ Not available

^c^ The proportional hazard regression
